# Elastic Properties of Defective 2D Polymers from Regression Driven Coarse-Graining

**DOI:** 10.1021/acs.jctc.5c01339

**Published:** 2025-10-23

**Authors:** David Bodesheim, Alexander Croy, Gianaurelio Cuniberti

**Affiliations:** † Institute for Materials Science and Max Bergmann Center for Biomaterials, 9169 TUD Dresden University of Technology, 01062 Dresden, Germany; ‡ Institute of Physical Chemistry, 9378 Friedrich Schiller University Jena, 07737 Jena, Germany; § Dresden Center for Computational Materials Science (DCMS), TUD Dresden University of Technology, 01062 Dresden, Germany

## Abstract

Two-dimensional polymers (2DPs) are an interesting class of polymers due to their reticular synthetic assembly, which make them an ideal platform for designing materials with specific target properties. Predicting and understanding their elastic behavior is crucial for their application. However, a realistic calculation of their properties remains computationally challenging due to the ubiquitous presence of defects in synthesized 2DPs. Here, we introduce a coarse-graining (CG) approach based on elastic beams called *MikadoRR* with parameters extracted from a simple regression-based fitting. This approach allows us to accurately calculate the elastic properties of defective 2DPs up to the microscale. Furthermore, we show that design principles of 2DPs for tailored elastic properties can be derived from this CG model.

## Graphical abstract

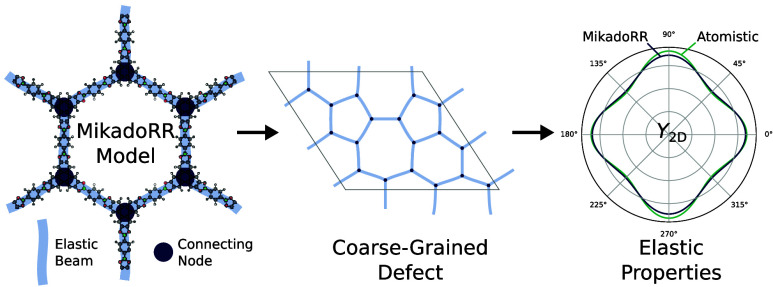

## Introduction

Two-dimensional polymers (2DPs) are periodic networks created by regularly linking monomers in-plane.^1,2^ Due to their structural diversity and unique properties, numerous applications have been explored such as gas separation^3^ and storage,^4^ membranes,^5^ sensing^6^ or energy storage.^7^ For pristine systems, relevant properties such as the electronic structure or elasticity of individual 2DPs are standard calculations with various computational atomistic methods, such as density functional theory.^8–10^ However, challenges arise for instance due to the combinatorial complexity of the underlying reticular chemistry for computational screening of materials and their properties.^11–16^ Moreover, synthesized 2DPs are known to contain defects within their structure which significantly influence the material’s behavior, such as the band structure,^17^ charge transport^18^ or the elastic properties,^19^ and 2DPs can be even be engineered to contain specific defects for tailored functionality.^20–22^ With the typically large unit-cell size of 2DPs, systems with defects or grain boundaries can lead to structures with ten-thousands of atoms, making property calculations with atomistic methods challenging if not impossible. Here, coarse-graining (CG) defective 2DPs provides a promising option. Only a few CG approaches have been applied for framework materials, such as metal–organic frameworks. In most cases, systems are represented by vertices of the framework connected by (a series) springs or beads.^9,23–27^ To achieve satisfactory results with these kinds of CG model, it is typically necessary to include multiple beads or connected springs, significantly increasing the parameter space. In our previous work, we discussed how the elasticity of 2D framework materials can be inferred from their building blocks by introducing CG models, such as a model based on simple springs (bond and angle force field (BAFF)) or a model using elastic beams (*Mikado* model).^28^ In this work, wedevelop a more general CG approach which treats linkages as elastic beams, encoding the necessary physical behavior into the model without drastically increasing the model’s complexity and resulting in transferability to defective systems. Correspondingly, an augmented Mikado CG model is used as a surrogate model to be trained via regression methods, which we term *MikadoRR*. We probe its generalizability and applicability for the calculation of large-scale defective systems and compare it to the simpler BAFF model. Finally, we show that due to the physics-inspired approach of the regression-driven MikadoRR model, we can derive qualitative design principles for the molecular building blocks to achieve tailored elastic properties of 2DPs.

## Methods

### MikadoRR Model

The *MikadoRR* model (see Figure 1a) extends our previous CG model used for 2D covalent organic frameworks^28^ which was inspired by *Mikado* models for the description of random polymer networks.^29–31^ Framework materials such as covalent organic frameworks or metal–organic frameworks can be represented by periodic nets, by replacing molecular building blocks at the core region by vertices and linker region by the edges of the net.^32^ In the *MikadoRR* model, the edges of such a net are represented by elastic beams which exhibit in-plane bending as well as compression and are connected at the vertices. The total energy of such an elastic net arises from contributions of the deformation of the elastic beams *b* themselves ( $H_{b}$ ) and the energy arising from the angle deviation between the beams at one vertex *m* ( $H_{m}$ )­

1 $$H_{\mathrm{MikadoRR}}=\sum_{b}H_{b}+\sum_{m}H_{m}+c_{7}$$

Here, *c*_7_ is a fitted offset parameter of the energy.

**Figure 1:**
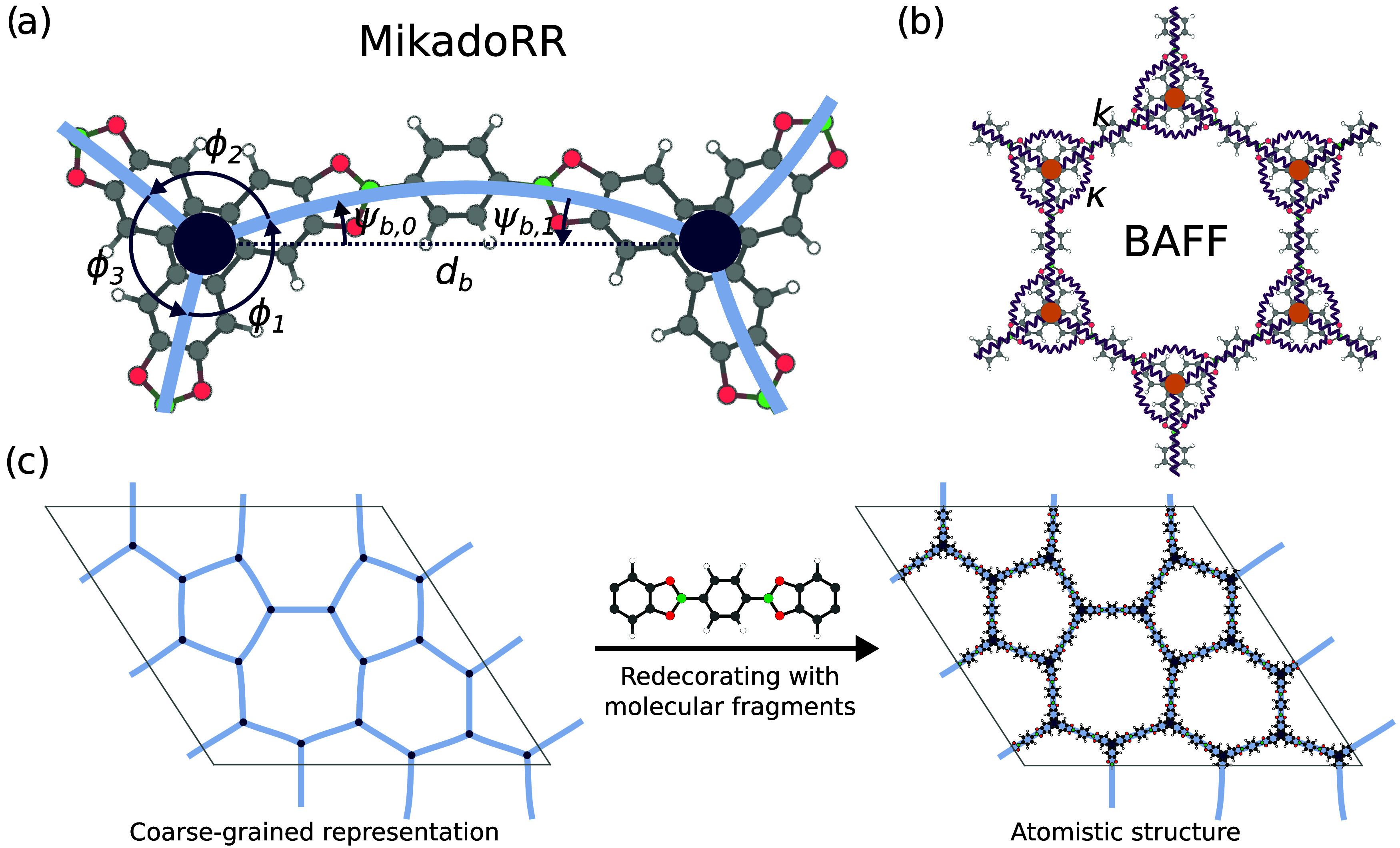
(a) Representation of the *MikadoRR* model with vertices (darkblue) connected by elastic beams (light-blue). (b) Representation of the bond and angle force-field (*BAFF*) with vertices (orange) connected by springs (purple) including angular springs. (c) Redecoration functionality which allows to translate a CG model into an atomistic structure by inserting and arranging molecular fragments.

Qualitatively, $H_{b}$ represents the elasticity of the linkages by including the compression of the beams through the distance between the vertices *d*_*b*_ and the in-plane bending through the angles ψ_*b*,0_ and ψ_*b*,1_ which are the angle of the outgoing beam at the incident vertex 0 and of the incoming beam connected to vertex 1, respectively. This yields the expression

2 $$H_{b}=c_{1}d_{b}+c_{2}d_{b}^{2}+c_{3}\psi_{b,0}^{2}+c_{4}\psi_{b,1}^{2}+c_{5}\psi_{b,0}\psi_{b,1}$$

The parameters *c*_1_ and *c*_2_ are an extension of the stretching force constant of our previous *Mikado* model (eq 6 in^28^) and related to the linear compressional rigidity of a linker molecule. The three parameters *c*_3_, *c*_4_, *c*_5_, *c*_6_ arise from the effective in-plane bending force-constant of an elastic beam from our previous *Mikado* model (eq 9 in^28^), which is comparable to an in-plane linker bending rigitidy of a linker molecule. The flexibility of the core region of a framework material is accounted for by $H_{m}$ using the angle ϕ_*mn*_ between the outgoing beams *n* at a vertex *m*,

3 $$H_{m}=c_{6}\sum_{n}^{3}\phi_{\mathrm{mn}}^{2}$$

The parameters *c*_1_–*c*_7_ are obtained from atomistic calculations of the deformation energies using a ridge-regression (RR) procedure, hence the name *MikadoRR*.

### BAFF Model

The simpler model *BAFF* was previously introduced in ref 28. and is depicted in Figure 1b. It depends only on two parameters, the stretching force constant *k* and the bending force-constant κ

4 $$H_{BAFF}=\frac{1}{2}\sum_{\mathrm{ij}}k{\left(d_{\mathrm{ij}}-d_{\mathrm{ij},0}\right)}^{2}+\frac{1}{2}\sum_{\mathrm{ijl}}\kappa {\left(\cos\left(\phi_{\mathrm{ijl}}\right)-\cos\left(\phi_{\mathrm{ijl},0}\right)\right)}^{2}$$

Here, *d*_*ij*_ is the distance between the neighboring vertices and ϕ_*ijl*_ is the angle spanned by vertices *i* and *l* neighboring vertex *j*.

The two force constants can be directly obtained from the 2D bulk (*B*_2D_) and the shear modulus (μ_2D_) for hexagonal systems^28^

5 $$k=\sqrt{3}B_{2D}$$

6 $$\kappa ={\left(\frac{1}{\sqrt{3}\mu_{2D}}-\frac{1}{2\sqrt{3}B_{2D}}\right)}^{-1}\frac{d_{\mathrm{ij},0}^{2}}{9}$$

Therefore, inherent bending of the linkages is not taken into account.

### Computational Details

The *MikadoRR* and *BAFF* are implemented as atomic simulation environment (ASE)^33^ calculators that evaluate energies, forces and numerical stresses. In the current implementation, all structures are strictly 2D, which approximates the typical confinement effects due to the deposition of 2DPs on substrates. Furthermore, the CG structure must be of periodic nature and have a coordination number equal to three. As masses are not part of the energy expression in both CG models, dynamic treatment with thermal effects is currently not available, even though it is possible in principle to assign masses and therefore a kinetic energy term and torque to the energy expression.

The CG models are fit to data of the pristine 2DP which in principle enables generating training data from accurate ab initio atomistic data on single unit cells and subsequently applying the CG models for large-scale simulations. However, to evaluate the performance of the models in large-scale defective systems which can easily exceed 20000 atoms, we train and benchmark the CG models to a simple force field (universal force field (UFF)^34^) which has been applied to 2DPs and framework materials before.^16,35–37^

We use an implementation of UFF within our in-house code *structure2lammps* (https://github.com/CoMeT4MatSci/structure2lammps)^38^ that automatically parametrizes a given atomistic structure. Calculations are performed with the Large-scale Atomic/Molecular Massively Parallel Simulator (LAMMPS)^39^ (version 2023.8.2.3.0) addressed via an ASE calculator implemented in *structure2lammps* in which all atoms for the simulations were constrained to remain in-plane during the relaxations.

Geometry optimizations are performed until the force on individual atoms/vertices is less than 0.001 eV/Å. For the in-plane cell-relaxation the in ASE implemented *FrechetCellFilter* is used.

For the *MikadoRR* model, first, the atomistic pristine system is isotropically strained (−1.0% to 1.0% strain) as well as sheared (−4.0% to 4.0% strain) yielding 41 strained structures. Then, the energies of the strained structures are calculated and the features *d*_*b*_, ψ, ϕ extracted. Finally, a ridge-regression (RR) with cross-validation as implemented in scikit-learn^40^ yields the fitted parameters *c*_1_–*c*_7_. A detailed description of the regression process is provided in the Supporting Information. It should be highlighted that no information about defects is included in the training data and hence we rely on the generalizability of this model for further calculations.

As this training data contains isotropically strained and sheared structures, *B*_2D_ and μ_2D_ are directly obtained from this data through equation of state fitting the energy and area or strain (see Supporting Information for details). From this, BAFF is parametrized using eq 5 and 6.

Elastic moduli are obtained by calculating the full 2D stiffness tensor based on the central difference approximation to the derivative of the stress tensor. Here, the structure is strained along different directions with ±0.4% strain, and the stress tensor is calculated after geometry relaxation. From this, the Voigt stiffness tensor is constructed from which other elastic properties are derived. This procedure is based on the implementation in the Computational 2D Materials Database.^41,42^ The radial dependence of the moduli is calculated and plotted with the ELATE code.^43^

To perform atomistic reference calculations of the CG structures, we implemented an automatic redecoration functionality which transforms a CG representation to the atomistic structure by placing, rotating and scaling molecular fragments accordingly (see Figure 1c and Supporting Information for details.

## Results

In the following sections, we evaluate the transferability and feasibility of the *MikadoRR* and BAFF model toward defective systems. We compare the defect formation energies and elastic properties with atomistic calculations. As a model compound for this, COF-5 will be used, which is one of the earliest and most studied covalent organic frameworks.^44^ The chosen defect and grain boundaries contain different ring sizes, which is commonly observed in polycrystalline 2DPs with honeycomb topology.^45,46^

### Defect Formation Energy

For the evaluation of defect formation energies, it is convenient to choose a defect that is generated without changing the number of atoms compared to the pristine structure, as the chemical potential stays constant. Such a defect is the well-known Stone–Wales (SW) defect which is generated by rotating two vertices by 90°, resulting in a defect with two five- and two seven-membered rings as visualized in Figure 2a. Throughout the analysis, we use the following naming scheme for the SW defect concentration: *n* × *n* SW defect, which means that based on the primitive unit cell of the pristine system, an *n* × *n* supercell is generated with one introduced SW defect.

**Figure 2:**
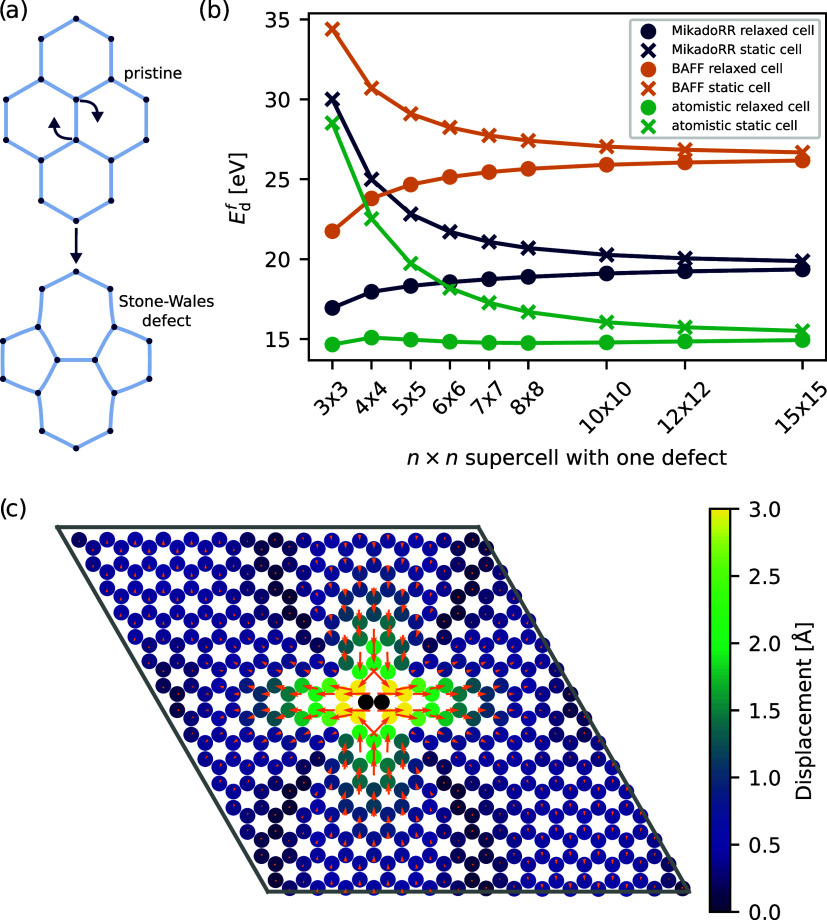
(a) Transformation from a pristine structure to a Stone–Wales defect. (b) Comparison of the SW defect formation energy for COF-5 for different defect concentrations. (c) Displacement of the CG cores in the MikadoRR model of a 15 × 15 SW defect compared to the pristine structure for a static cell. The orange arrows indicate the displacement vectors and are scaled by a factor of 10 for better visualization. The central two CG cores of the SW defect are not being considered and shown in black.

Figure 2b shows the relationship between the defect formation energy *E*_*d*_^*f*^ = *E*_defect_ – *E*_pristine_ and different defect concentrations for *BAFF* and *MikadoRR* and a comparison with atomistic simulations. Two cases are probed: first, including in-plane cell relaxation, and second, using a static cell, where the dimensions of the static cell are those of the pristine system. In both cases, *E*_*d*_^*f*^ converges to the same limiting value for low defect concentrations, which is an effectively isolated defect energy. However, if cell-relaxation is permitted, *E*_*d*_^*f*^ increases with a lower defect concentration. In the low-concentration regime, the SW defect is confined by a surrounding pristine region, which forces the SW into a locally energetically undesirable state in which it cannot fully relax. This effect is clearly visible in the displacements of the CG cores around the SW defect in Figure 2c. The sites in proximity to the SW defect are strongly displaced, but the displacement quickly decays so that regions further away from the SW defect are effectively not affected by its insertion. Visualizing the per-site energies clearly shows the effect of the strong local displacements where the per-site energies rapidly decrease away from the SW defect (see Figures S2 and S3). For higher defect concentrations, this effect is less pronounced as the SW defect dictates the geometry of the structure and the cell. For a high defect-concentration, the defect is free to deform the cell and disperse local stresses. In the case of a static cell, this is not possible, and hence *E*_d_^*f*^ increases in the high defect-concentration regime.

In general, *BAFF* highly overestimates the defect energy, although it shows a similar trend regarding the defect concentration. In the *MikadoRR* model, there are more degrees of freedom to distribute the local stresses, resulting in a better approximation to the atomistic structure. It should be noted that *BAFF* and *MikadoRR* agree almost perfectly with each other regarding the CG site positions (with an average displacement with respect to each other of 0.036 Å, see Figure S1), meaning that not the geometric difference is responsible for the different defect energy, but the available degrees of freedom in the models themselves. Another indicator for this is the excellent agreement of cell parameters of the SW defect simulations of the CG models and the atomistic representation (see Figure S4).

### Elastic Properties

To probe the transferability of the CG models for the evaluation of elastic properties in defective systems, we calculated the full 2D stiffness tensor for various defects as well as defect concentrations. Defects introduce anisotropy into intrinsically isotropic hexagonal systems. Figure 3(a–d) depicts the radial dependence of the 2D Young’s modulus *Y*_2D_ of the highest possible concentration of a SW defect, a 585-defect and two types of 558 grain-boundaries,^47,48^ respectively. For all defects, *MikadoRR* is in very good agreement with the atomistic data, despite its parametrization from pristine structures. Reference calculations with density functional based tight binding show a similar behavior (see Figure S9). However, *BAFF* is mainly unable to reproduce this behavior and consistently underestimates the anisotropy. This becomes especially visible for the highly anisotropic SW defect in Figure 3a where *BAFF* predicts an almost isotropic behavior. This further indicates its shortcomings as a result of the few available degrees of freedom.

**Figure 3:**
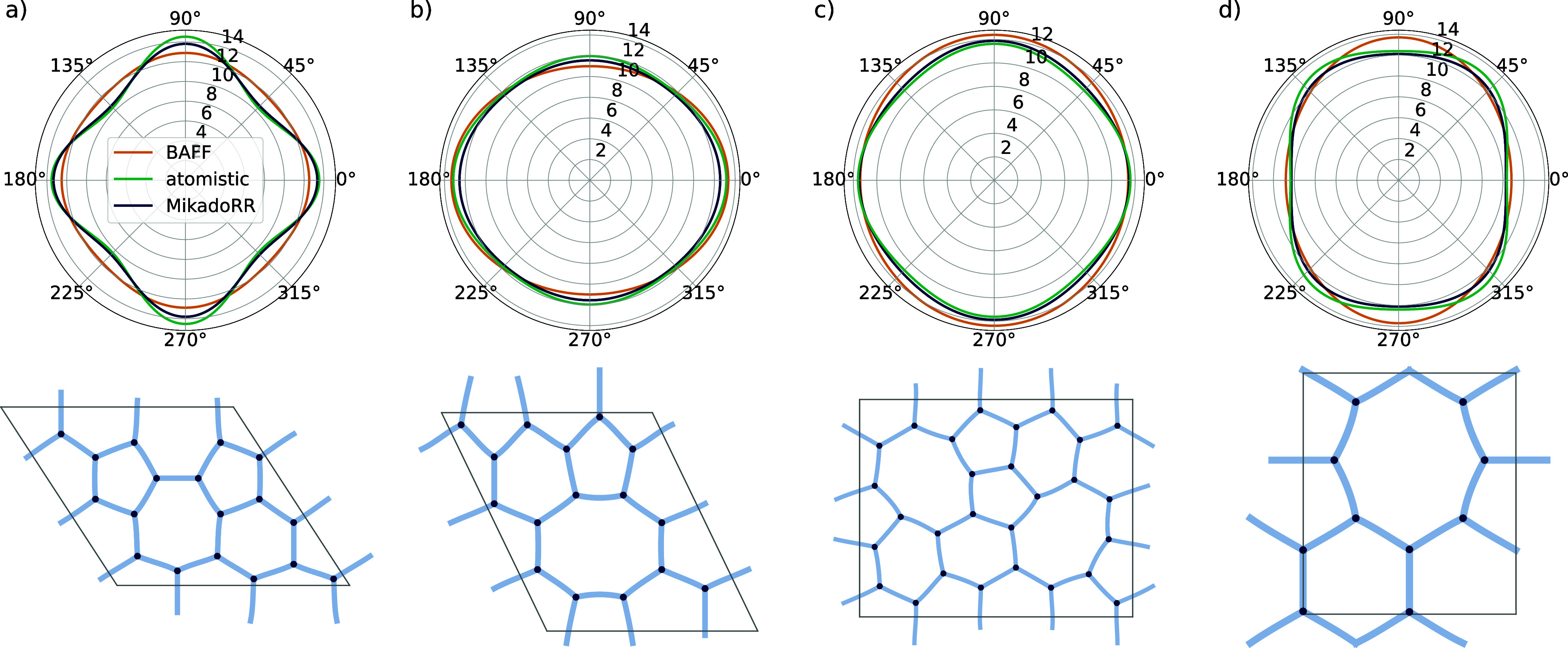
Comparison between *BAFF*, *MikadoRR* and the atomistic calculations for the radial dependence of the 2D Young’s modulus *Y*_2D_ in units of N/m for a (a) SW defect, (b) 585 defect, (c) 558 grain boundary type 1 and (d) 558 grain boundary type 2. A strong the deviation from a circular shape relates to elastic anisotropy. The bottom panels show the CG representation of the investigated defects of COF-5.

From the stiffness tensor, the 2D bulk modulus *B*_2D_ is calculated for different defect concentrations of SW defects, as depicted in Figure 4a. *MikadoRR* and *BAFF* have an almost identical behavior and both overestimate *B*_2D_ particularly for high defect concentrations. Nevertheless, the two models correctly predict an increase of *B*_2D_ for lower defect concentrations that converges toward the modulus of the pristine structure. The relatively good performance of *BAFF* compared to the previously discussed 2D Young’s modulus can be attributed to a low amount of bending of the linkers in isotropic compression, which is usually the weakness of *BAFF*. This is also the reason for its similar performance to *MikadoRR*, as the angular terms in eq 2 are small for isotropic compression resulting in energy contribution mainly from harmonic spring potentials of the beam compression.

**Figure 4:**
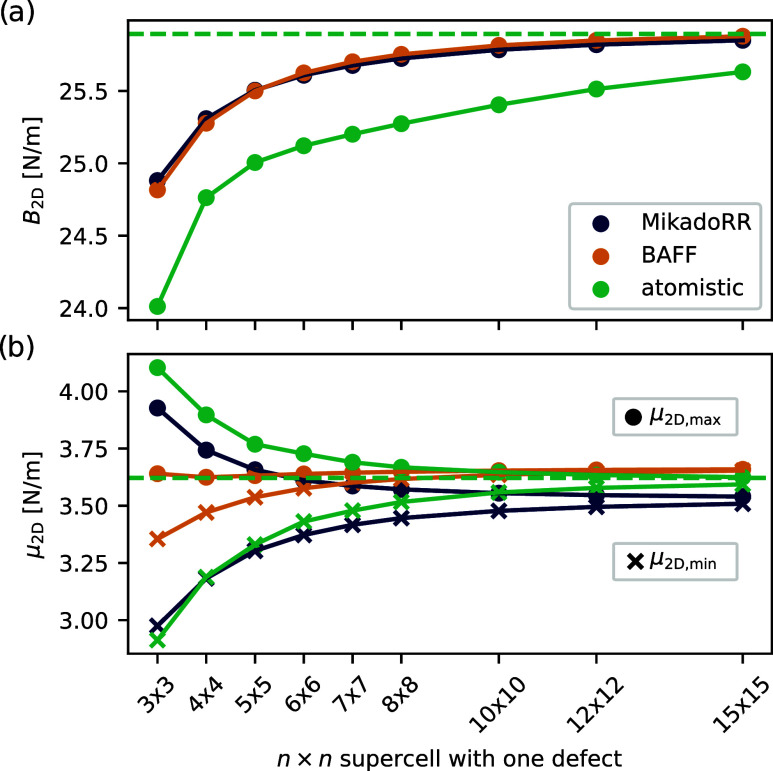
Dependency of the (a) 2D bulk modulus *B*_2D_ and (b) the minimum and maximum value of the radially dependent shear modulus μ_2D,min_ and μ_2D,max_ for different SW defect concentrations for *BAFF*, *MikadoRR* and atomistic calculations. The dashed horizontal line indicates the respective modulus for an atomistic pristine system.

However, if shearing contributions are present and therefore linker bending is important to consider, the limitations of *BAFF* become clear, for instance for the 2D shear modulus μ_2D_ of SW defects, as shown in Figure 4b. Here, the difference in the maximum and minimum value of μ_2D_ is severely underestimated in *BAFF. MikadoRR* is better suited to capture anisotropy. Nevertheless, both models converge for low concentrations close to μ_2D_ of the pristine structure.

### *MikadoRR* Model for the Rational Design of 2DPs

The parameters in the *MikadoRR* model are qualitatively related to the elastic properties of the building blocks of 2DPs. For instance, *c*_1_ and *c*_2_ correspond to the compressional stiffness of the linkages, *c*_3_, *c*_4_ and *c*_5_ are related to the in-plane bending rigidity of the linkages and *c*_6_ can be interpreted as the in-plane bending rigidity of the core region. By artificially changing these parameters in a trained model, their influence on properties of the 2DP is systematically investigated and hence qualitative design rules for 2DPs derived. In the following, we use the parametrized *MikadoRR* model of COF-5 and focus on the influence of the in-plane bending rigidity of the linker and core on several properties, such as the shear modulus of a pristine system μ_2D,prist_, the defect formation energy of a 15 × 15 SW defect *E*_*d*,15 × 15_^*f*^ and the anisotropy of the 2D Young’s modulus of a 3 × 3 SW defect *A*_*Y*,3 × 3_.

Figure 5a depicts how increasing the rigidity of either the cores or linkers yields a higher shear modulus of the pristine 2DP μ_2D,prist_. This implies that in a sheared structure mostly the more flexible part of the 2DP is bent, i.e., in the case of a flexible (rigid) linker and rigid (flexible) core, the linker (core) is bent in a sheared pristine structure while the core (linker) stays rigid, as visualized in Figure S13.

**Figure 5:**
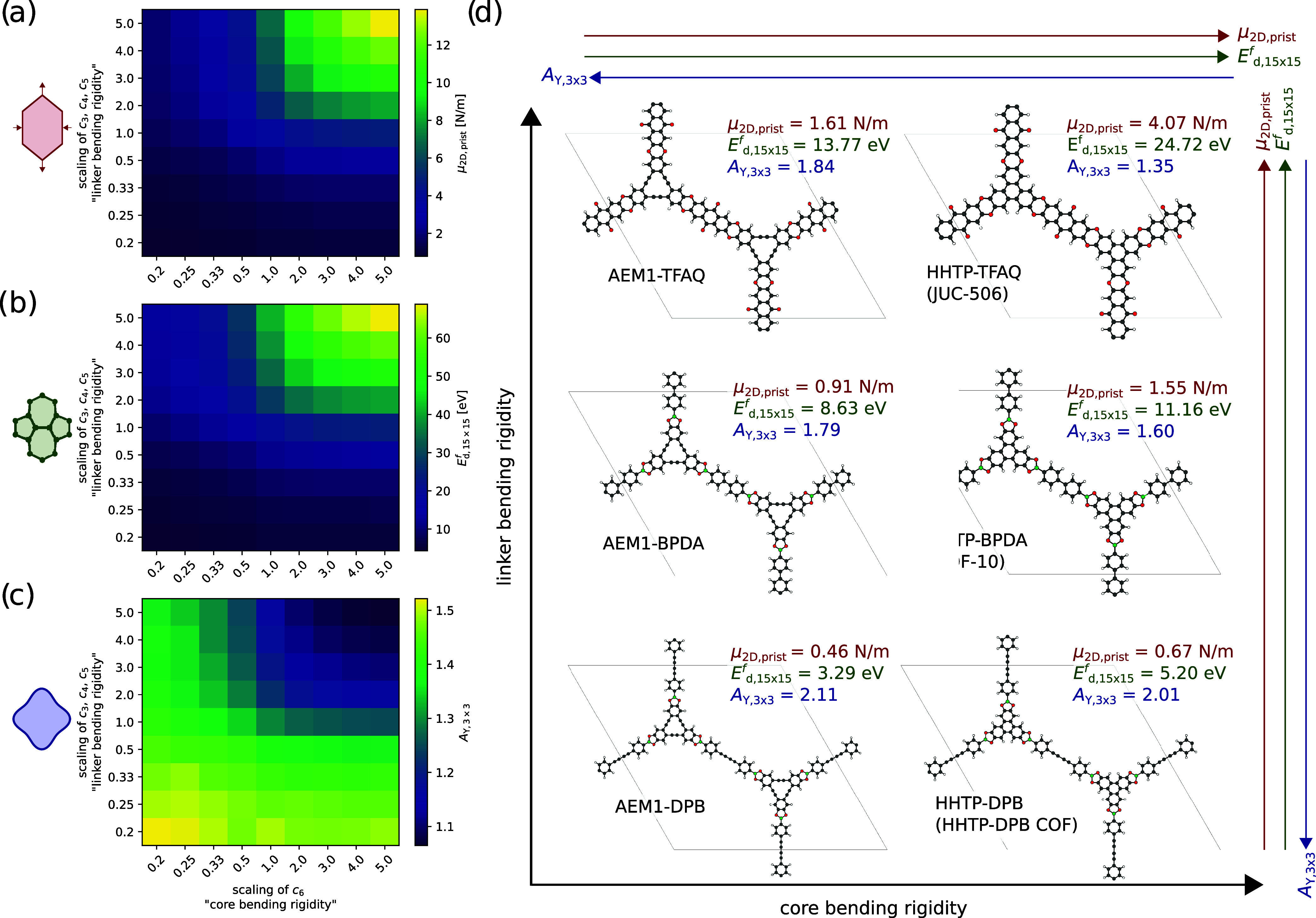
Variation of the (a) 2D shear modulus μ_2D, prist_ for a pristine system, (b) defect energy *E*_*d*,15×15_^*f*^ in a 15 × 15 SW defect, (c) anisotropy of the 2D Young’s modulus defined by $A_{Y,3\times 3}=\frac{Y_{2D,\max}}{Y_{2D,\min}}$ in a 3 × 3 SW defect. The x- and *y*-axis represent the scaling of the parameter *c*_6_ (corresponding to the core bending rigidity) and the parameters *c*_3_, *c*_4_ and *c*_5_ (corresponding to the linker bending rigidity) in the *MikadoRR* model. (d) Overview of different COFs and their elastic properties. The names are based on the core-linker molecule combination. If the COF is known in literature, the common name is written in parentheses.

Next, the defect formation energy of a 15 × 15 SW defect *E*_*d*,15×15_^*f*^ was investigated, as visualized in Figure 5b. This is an important property, as it is the energy of an isolated defect within an otherwise pristine structure and qualitatively describes the energetic likelihood of a thermodynamically driven defect. A stronger in-plane bending rigidity of either the core or the linker increases the defect formation energy. Since the previously discussed shear modulus exhibits a similar trend, μ_2D, prist_ and *E*_*d*,15×15_^*f*^ are strongly correlated (see Figure S14).

Finally, the influence on the elastic anisotropy of the 2D Young’s modulus in a 3 × 3 SW defect *A*_*Y*,3×3_ is explored in Figure 5c. The more rigid the cores or linkers regarding their in-plane bending, the lower the elastic anisotropy in a SW defect. Therefore, there is a strong inverse correlation between μ_2D,prist_ and *A*_*Y*,3×3_ (see Figure S15).

The investigated trends show that for the targeted design of the elastic properties a freedom of design regarding the core and linker properties is present. Based on the results, μ_2D,prist_ as a single parameter is enough to determine qualitative (but not quantitative) trends for derived properties of defective systems. As *BAFF* is directly parametrized from the bulk and shear modulus, we investigate if *BAFF* alone is sufficient to predict these properties. We parametrize new *BAFF* models based on the *MikadoRR* models by calculating the bulk and shear modulus of a pristine system with the *MikadoRR* model and use these for new *BAFF* models. The resulting models show that the less rigid the cores and the more rigid the linkers with respect to their bending, the better *BAFF* coincides with *MikadoRR* to describe the SW defect formation energies (see Figure S16) as in this case bending of linkers is not strongly prevalent. Furthermore, *BAFF* always underestimates the anisotropy and as the anisotropy generally reduces with stiffer linkers and cores, *MikadoRR* is more similar compared to *BAFF* in terms of elastic properties for stiffer materials (see Figure S17 for a comparison of the anisotropy and Figure S18 for a comparison of *Y*_max_ between the two models). Hence, *BAFF* predicts correctly the trends qualitatively, but not quantitatively.

To test wether the design principles can be transferred to atomistic systems, we chose 2DPs with a combination of soft and rigid cores and linkers. The cores and linkers are based on precursors commonly used in literature for COF synthesis. As an example of a rigid core, we chose 2,3,6,7,10,11-hexahydroxytriphenylene (HHTP), which is a commonly used monomer unit used for instance in the synthesis of COF-5,^44^ COF-10,^49^ JUC-506 ^50^ or the HHTP-DPB COF.^51^ As soft core, an arylene-ethynylene macrocycle commonly referred to as AEM-1 (or DBA[12]) was selected which is a monomer for COFs such as DBA-COF 1 ^52^ or Py-DBA-COF 1.^53^ The linker molecules ranging from rigid to soft were inspired from the monomers of JUC-506,^50^ COF-10,^49^ the HHTP-DPB COF^51^ which are 2,3,6,7-tetrafluoroanthraquinone (TFAQ), 4,4’-biphenyldiboronic acid (BPDA) and 4,4’-diphenylbutadiynebis­(boronic acid) (DPB) respectively.

Figure 5d shows an overview of the studied 2DPs with ranging core and linker bending rigidity. The atomistically calculated properties coincide with the previously discussed trends. It is clearly shown, that μ_2D, prist_ is a strongly deterministic quantity for other properties. By choosing the right combination of cores and linkers, we can rationally aim for specific elastic properties. For instance, AEM1-TFAQ has a soft core but a rigid linker and has similar elastic properties as HHTP-BPDA (COF-10) which has a rigid core and a softer linker. If, for example, the goal is a high defect formation energy, but one is constrained in the synthesis to a soft core such as AEM1, one could rationally interchange the linker for a more rigid one, such as in the case of AEM1-TFAQ. This principle of targeted design of elastic properties of 2DPs through a freedom of tuning either the elasticity of the core or linker region paves the way to rationally guided synthesis strategies.

## Conclusion

In summary, we introduced a regression-based two-dimensional few-parameter CG model based on elastic beams for 2D framework materials called *MikadoRR* and calculated theelastic properties for various defects and defect concentrations. We showed that the model, even though only fitted from a few strained pristine configurations, is in close agreement with atomistic results for elastic properties of defective structures and can be used for calculations of microscale-sized systems usually not attainable by accurate atomistic calculations. Furthermore, we compared this model to a simplistic spring-based model (*BAFF*) and showed that the description of bending linkages encoded in *MikadoRR* is crucial for an accurate evaluation of the elastic properties of 2DPs. Finally, we showed that based on the parameters of the *MikadoRR*, we can infer qualitative design rules for 2DPs regarding their elastic properties which can serve as a starting point for experimental validation.

In future work, the presented model can be extended in various ways to account for even more realistic calculations. For instance, as 2DPs are usually not completely confined to two dimensions, an additional description of out-of-plane bending is necessary for a complete representation of their elastic properties.^54^ This could be accounted for by adding out-of-plane angular contributions to the elastic beams. Furthermore, a connectivity of three is currently required which can be easily extended. Even though all simulations are static without the influence of temperature and thermal effects are known to influence the elastic properties of free-standing 2DPs monolayers,^55^ the results are still indicative of realistic material property trends.

We believe that the *MikadoRR* model can be instrumental for a systematic understanding and design of two-dimensional framework materials as well as a new approach of coarse-graining of these types of systems in the future.

## Supplementary Material



## Data Availability

The code for the CG models *BAFF* and *MikadoRR* are available at https://github.com/CoMeT4MatSci/cgf.^56^ The code for the force-field calculations is available at https://github.com/CoMeT4MatSci/structure2lammps.^38^ All other data for the reproduction of this study is available on Zenodo.^56^

## Notes

Additional plots for comparison between UFF, *MikadoRR*, and *BAFF*. Radial stiffness plots for different types of defects; comparison between DFTB and UFF; more detailed description, derivation and fitting procedure of *MikadoRR* and *BAFF* as well as the redecorating functionality (PDF)
