# Supplementary material for: Elastic Properties of Defective 2D Polymers from Regression Driven Coarse-Graining
Source: J Chem Theory Comput. 2025 Oct 23;21(21):11210–8. doi: 10.1021/acs.jctc.5c01339 (PMC12613322; doi:10.1021/acs.jctc.5c01339)
Supplement: Supplementary file 1 [file ct5c01339_si_001.pdf]

Supporting Information: Elastic properties of defective 2D polymers from regression driven coarse-graining

David Bodesheim,[1] Alexander Croy,[2] and Gianaurelio Cuniberti[1,3]

[1]*Institute for Materials Science and Max Bergmann Center for Biomaterials, TUD Dresden University of Technology, 01062 Dresden, Germany*
[2]*Institute of Physical Chemistry, Friedrich Schiller University Jena, 07737 Jena, Germany*
[3]*Dresden Center for Computational Materials Science (DCMS), TUD Dresden University of Technology, 01062 Dresden, Germany*

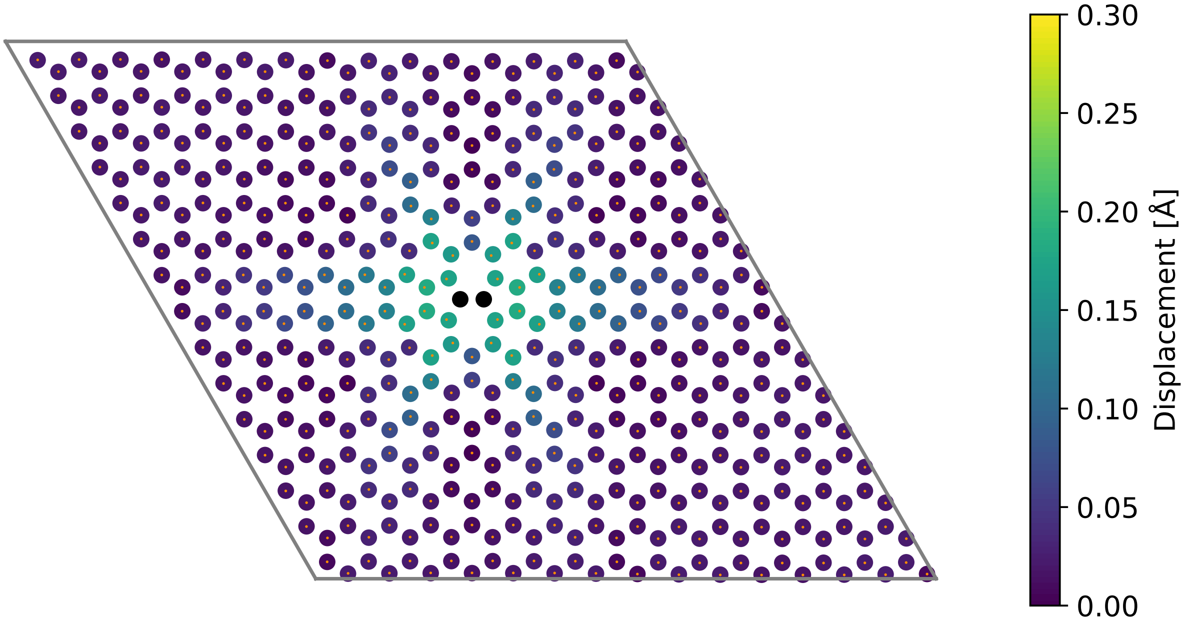

FIG. S1. Comparison between the displacement of the CG cores of a 15x15 SW defect in the *MikadoRR* model compared to *BAFF* model for a static cell. The orange arrows indicate the displacement vectors and are scaled by a factor of 10 for better visualization. The central two CG cores of the SW defect are not being considered and shown in black.

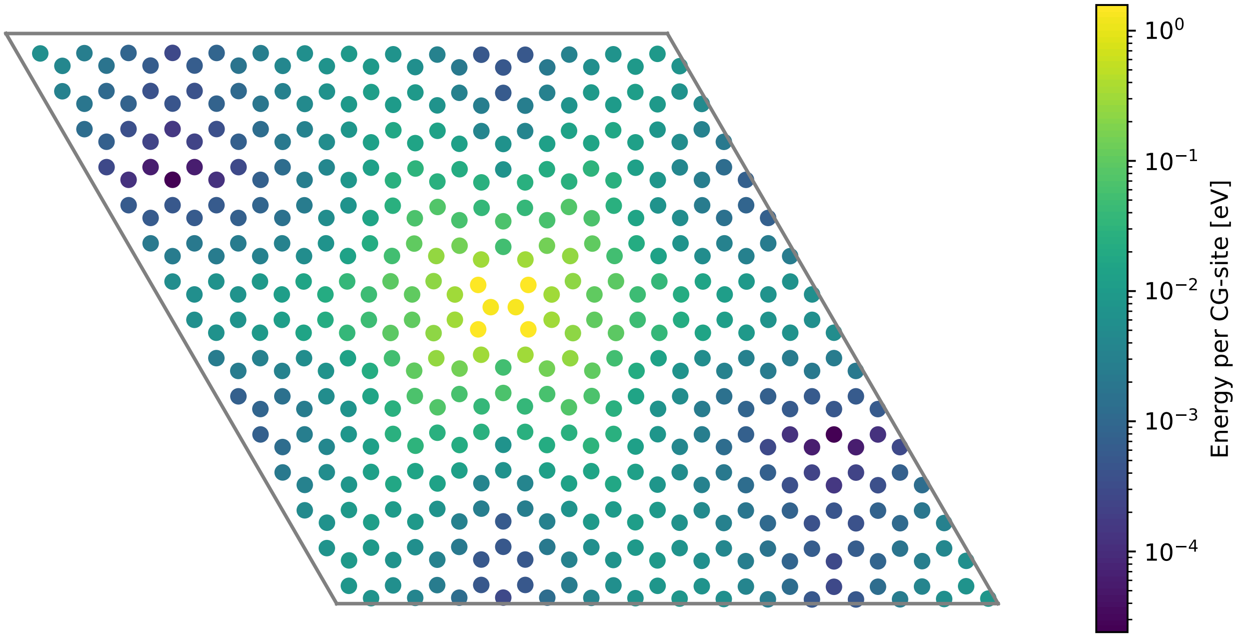

FIG. S2. Logarithmic per CG site energy of a 15x15 SW defect in the *MikadoRR* model. The site energies were approximated by splitting the energy contribution of each beam between two cores (eq. 2 in manuscript) equally between them and then adding the respective core flexibility contribution (eq. 3 in manuscript). The maximum site energy is 1.57 eV. 0 eV corresponds to the energy in the pristine system.

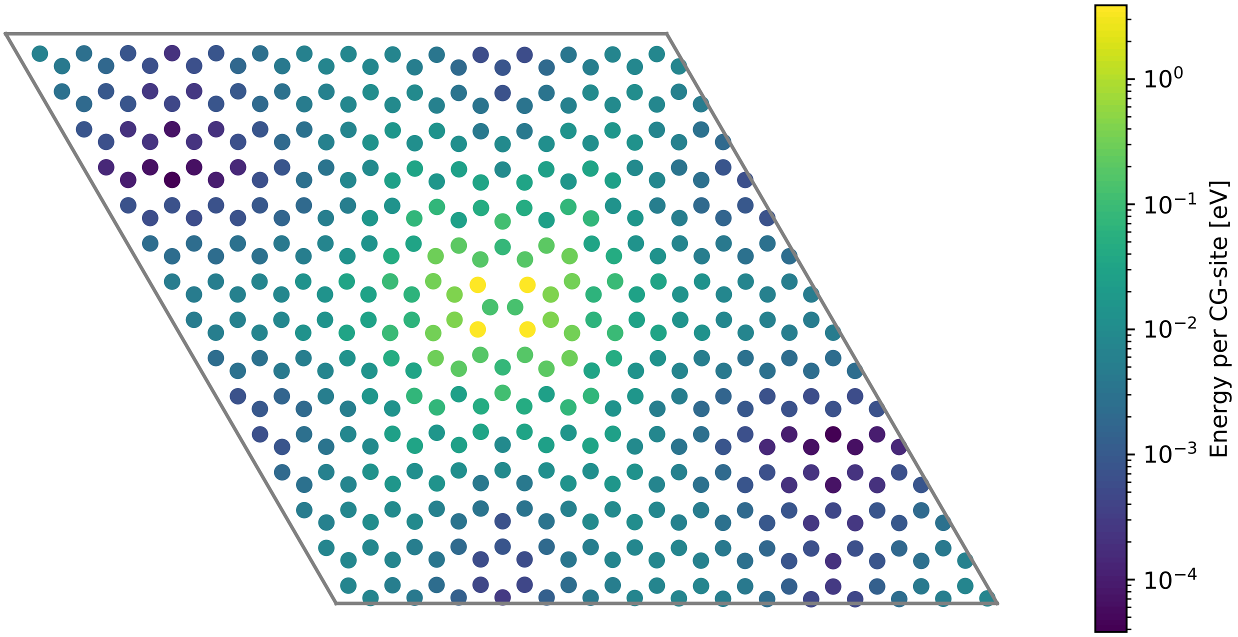

FIG. S3. Logarithmic per CG site energy of a 15x15 SW defect in the *BAFF* model. The site energies were approximated by splitting the energy contribution of each spring between two cores (first term in eq. 4 in manuscript) equally between them and then adding the respective core angular contribution (second term in eq. 4 in manuscript). The maximum site energy is 3.90 eV. 0 eV corresponds to the energy in the pristine system.

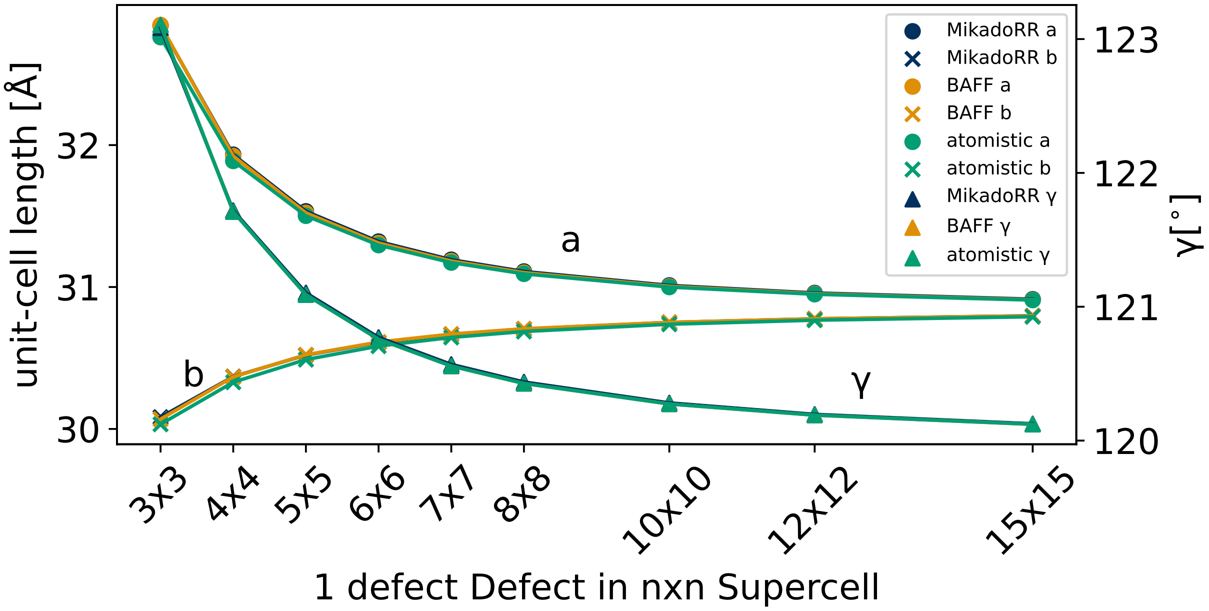

FIG. S4. Comparison of the cell parameters of the SW defect for COF-5 based on UFF for different defect concentrations. The unit-cell length is defined as the cell-length divided by n, where n is the $n \times n$ supercell.

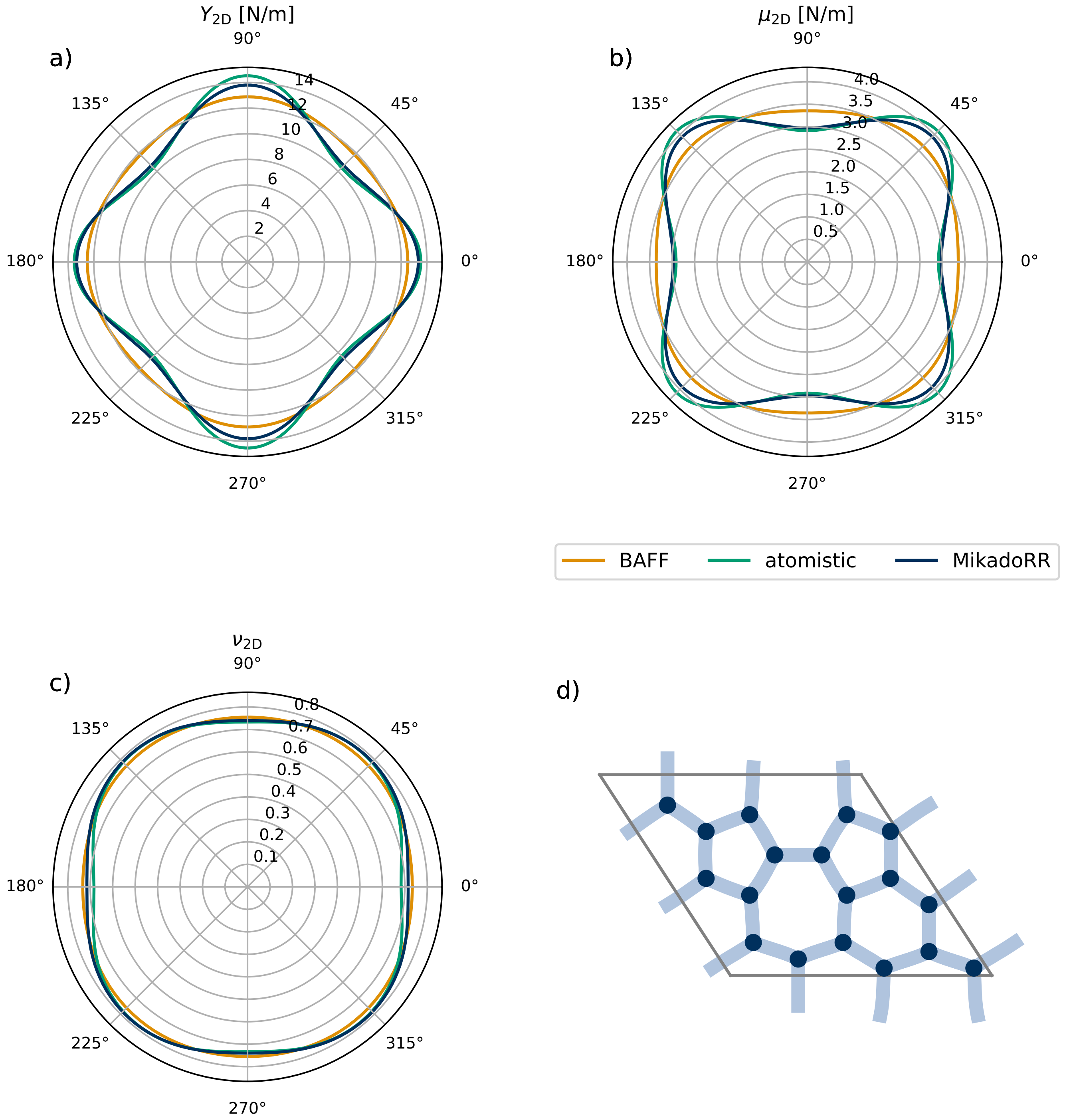

FIG. S5. Comparison between *BAFF*, *MikadoRR* and the UFF atomistic calculations for the radial dependence of the a) 2D Young's modulus, b) shear modulus and c) Poisson's ratio for a 3x3 SW defect of COF-5 shown in the coarse-grained representation in d).

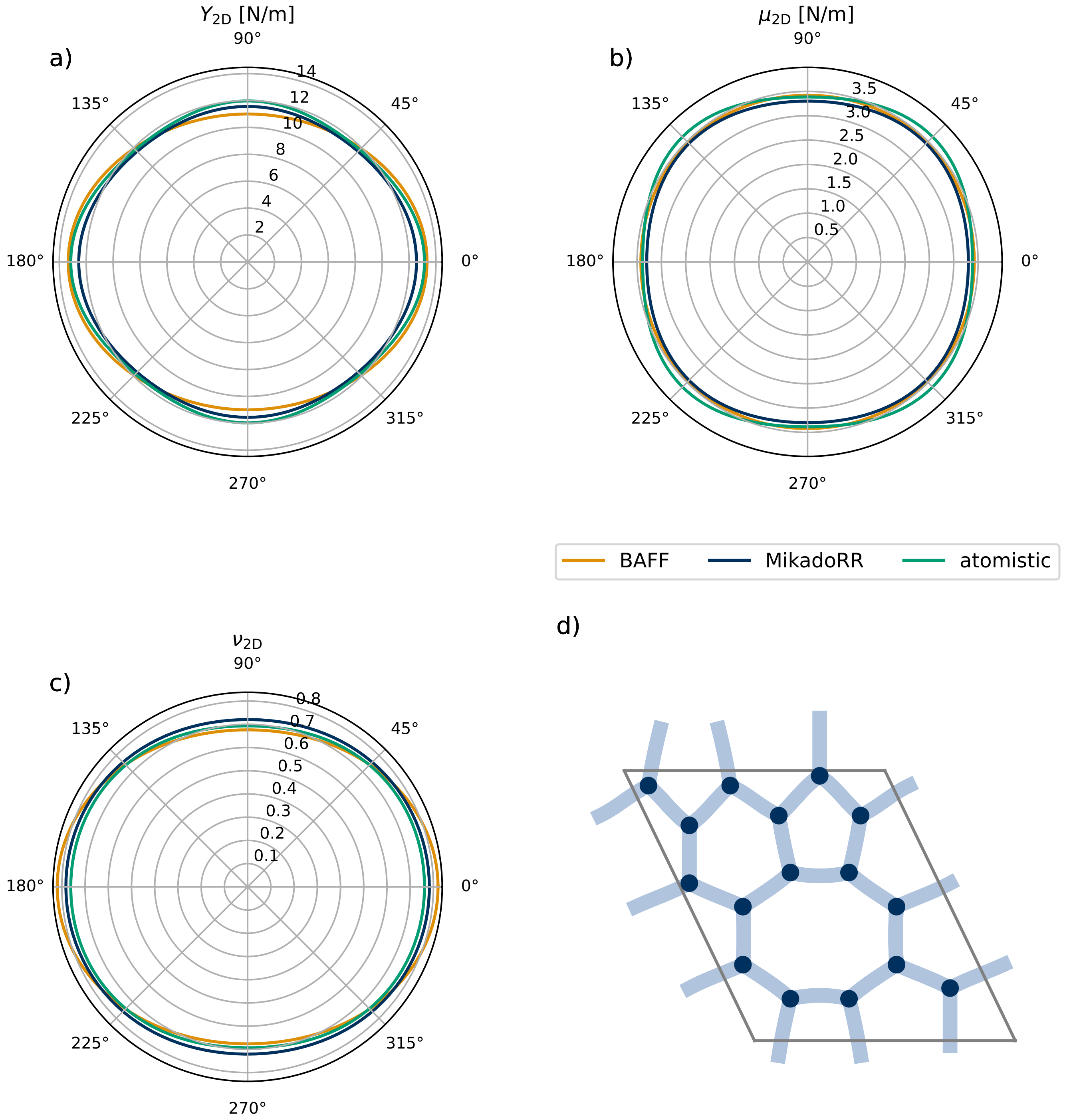

FIG. S6. Comparison between *BAFF*, *MikadoRR* and the UFF atomistic calculations for the radial dependence of the a) 2D Young's modulus, shear modulus and Poisson's ratio for a 3x3 585 defect of COF-5 shown in the coarse-grained representation in d).

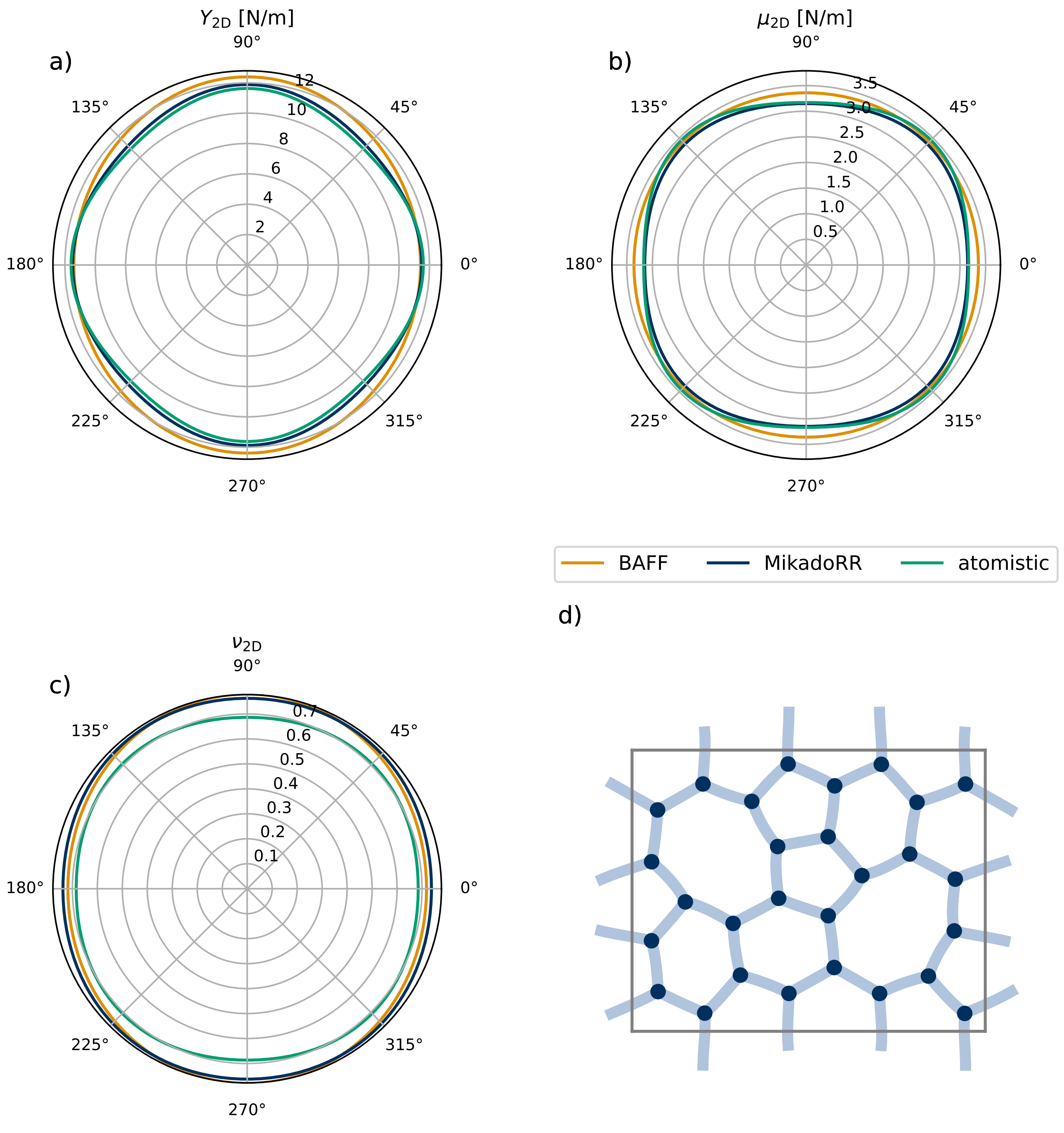

FIG. S7. Comparison between *BAFF*, *MikadoRR* and the UFF atomistic calculations for the radial dependence of the a) 2D Young's modulus, b) shear modulus and c) Poisson's ratio for a 558 grain boundary defect of COF-5 shown in the coarse-grained representation in d).

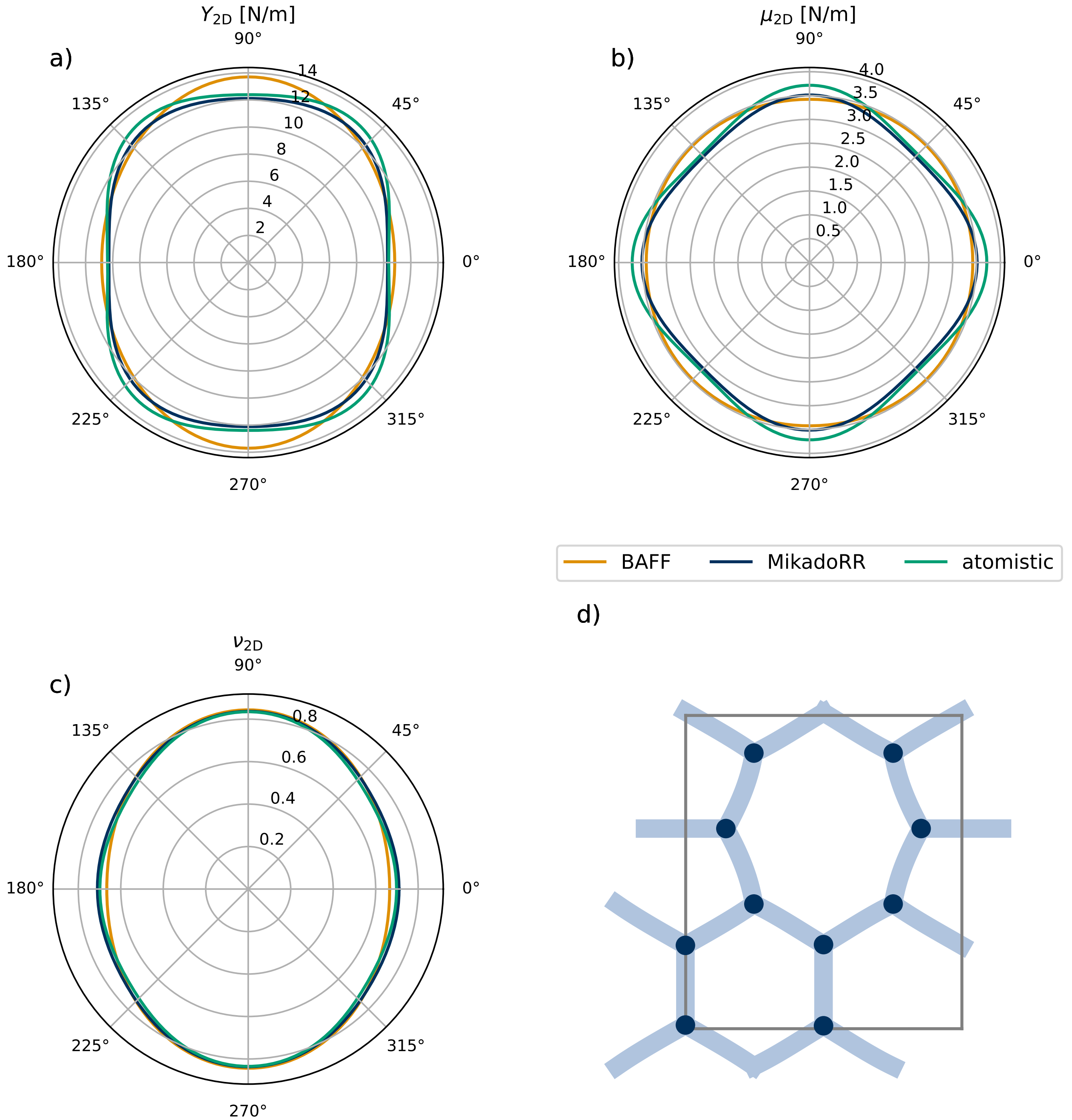

FIG. S8. Comparison between *BAFF*, *MikadoRR* and the UFF atomistic calculations for the radial dependence of the a) 2D Young's modulus, b) shear modulus and c) Poisson's ratio for a 558 grain boundary defect of COF-5 shown in the coarse-grained representation in d).

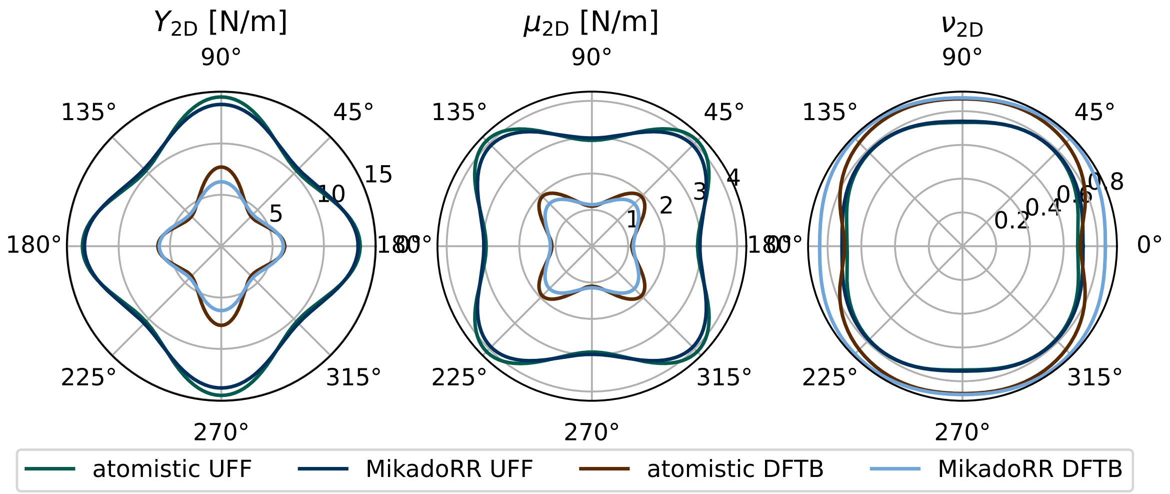

FIG. S9. Comparison between atomistic UFF, *MikadoRR* trained on UFF, atomistic density functional based tight binding (DFTB) and *MikadoRR* trained on DFTB calculations. Depicted is the radial dependence of the a) 2D Young's modulus, b) shear modulus and c) Poisson's ratio for a for a 3x3 SW defect. The DFTB calculations we performed with DFTB+[SI1, SI2] with the matsci parameter set [SI3] including SCC and DFT-D4 correction [SI4] with s6=1.0, s8=3.3157614, s9=1.0, a1=0.4826330,a2=5.3811976. The electronic Fermi-temperature was set to 50K for better convergence.

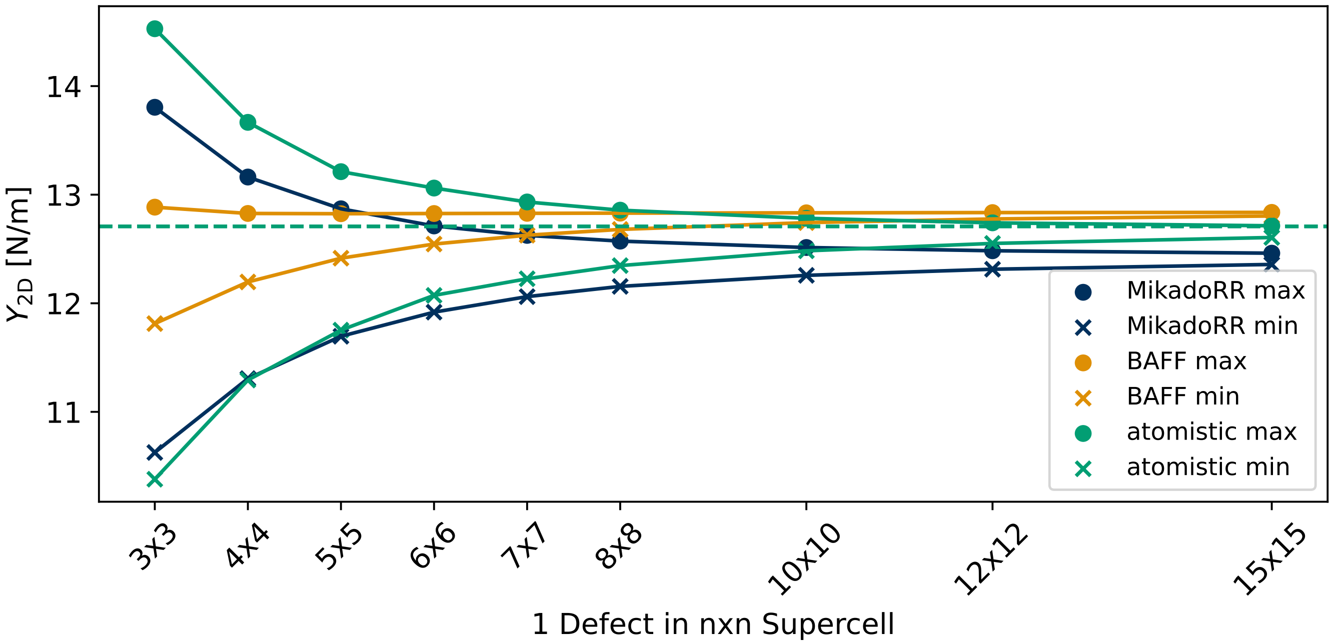

FIG. S10. Dependence of the minimum and maximum 2D Young's modulus for different SW defect concentrations for *BAFF*, *MikadoRR* and atomistic UFF calculations. The dashed horizontal line indicates the Young's modulus for a pristine system.

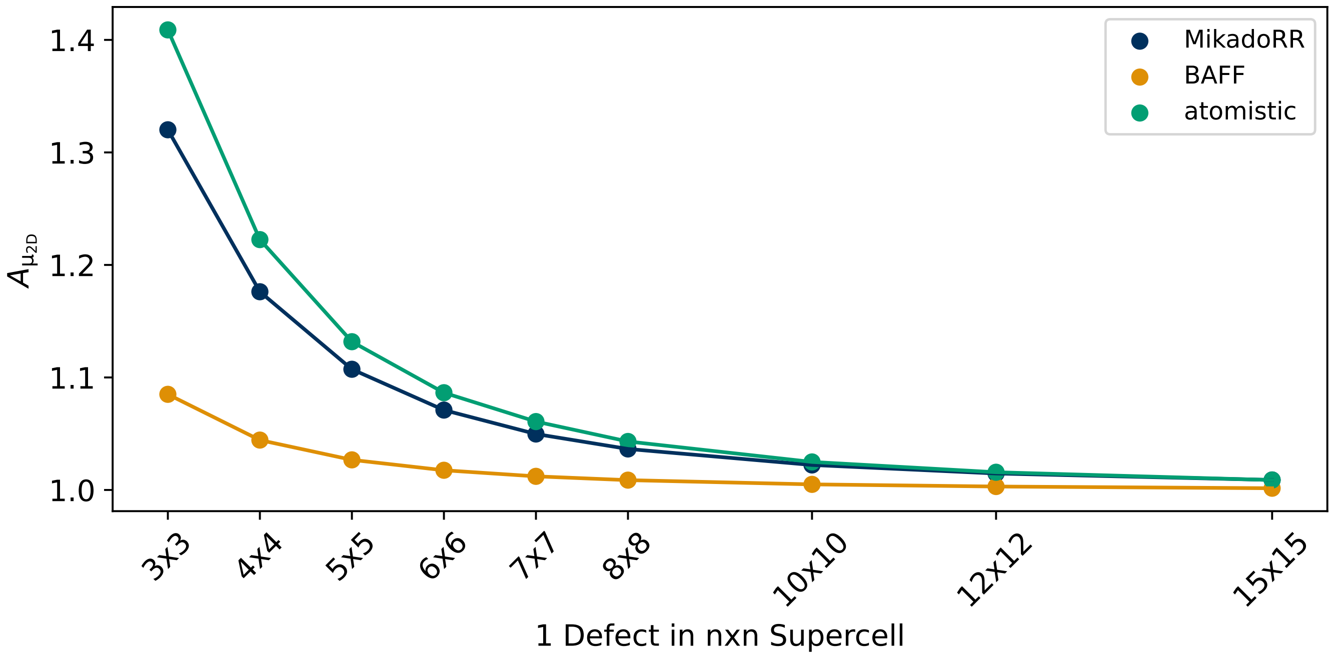

FIG. S11. Dependence of the anisotropy A of the shear modulus for different SW defect concentrations for *BAFF*, *MikadoRR* and atomistic UFF calculations.

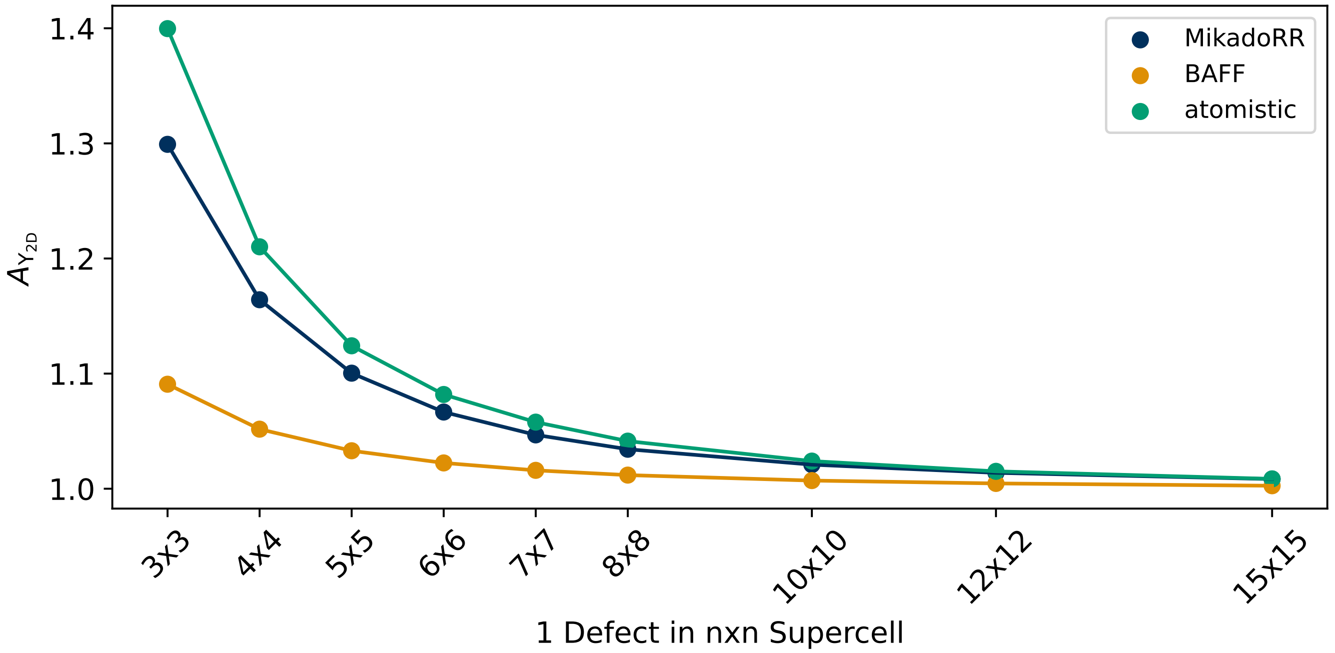

FIG. S12. Dependence of the anisotropy A of the Young's modulus for different SW defect concentrations for *BAFF*, *MikadoRR* and atomistic UFF calculations.

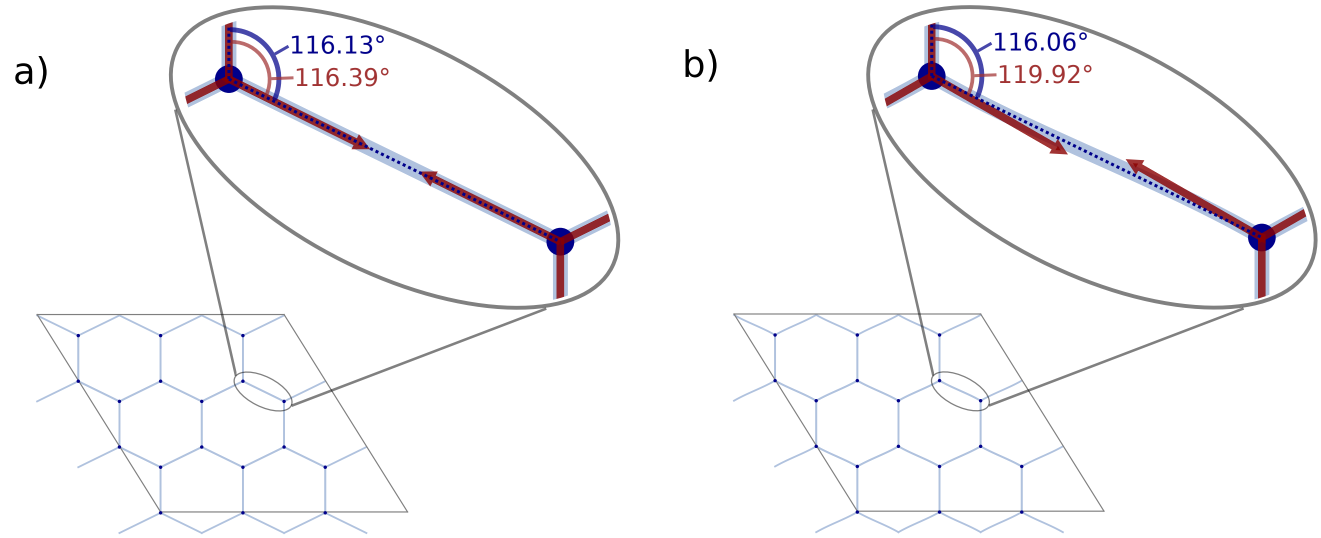

FIG. S13. Coarse-Grained representation of COF-5 during shearing (γ = 122.0°). The light blue beams indicate the elastic beams. In the insets, the red arrows represent the linker-site vectors. The dark blue dotted line indicates the vector between two neighboring cores. The blue angle indicates the angle between two neighboring cores. The red angles indicate the angle between linker-sites. a) shows the structure for a rigid linker (c_3, c_4, c_5 scaled by 5.0) and a flexible core (c_6 scaled by 0.2) and b) vice versa (c_3, c_4, c_5 scaled by 0.2 and c_6 scaled by 5.0).

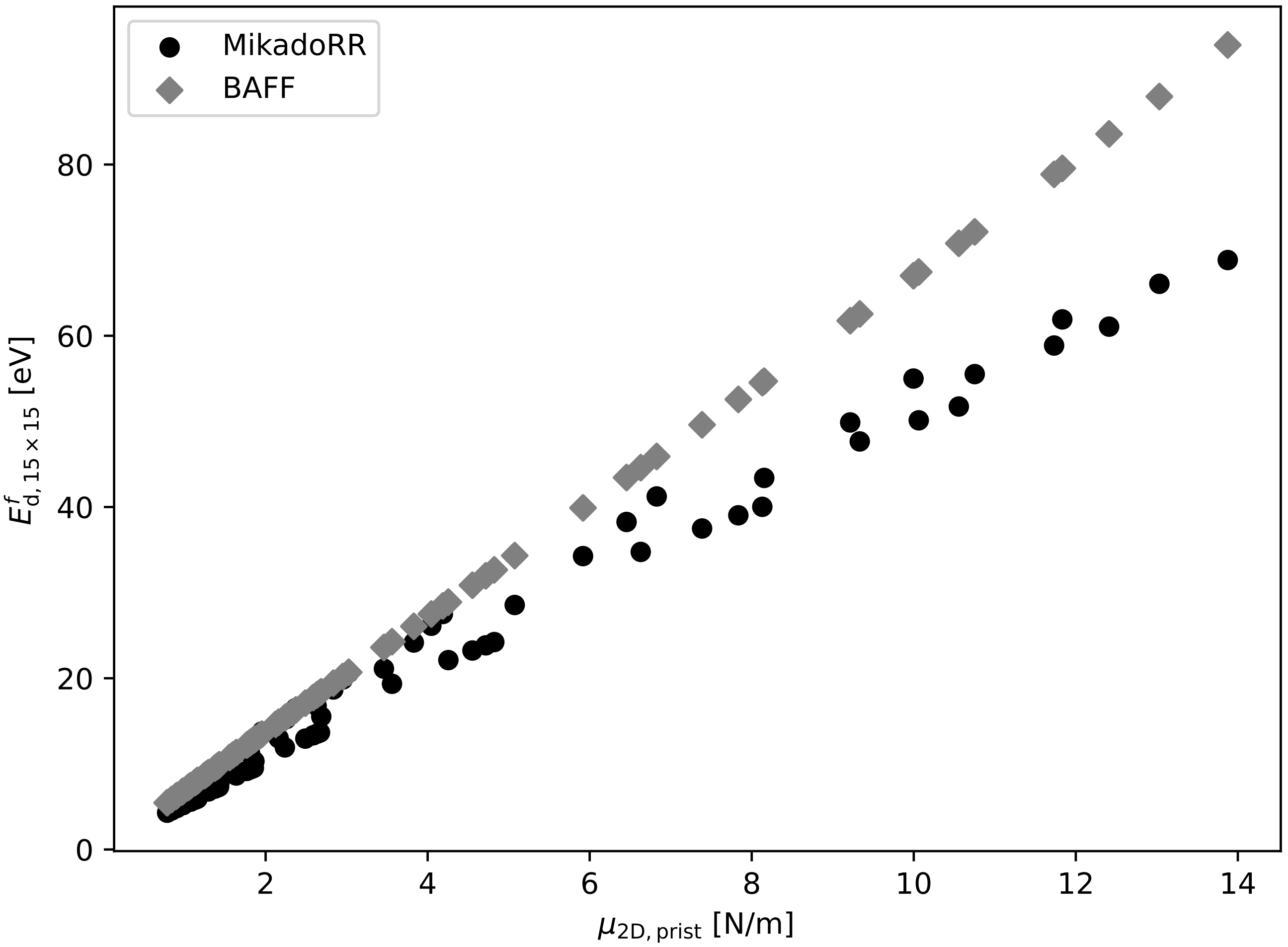

FIG. S14. Correlation between the shear modulus of the pristine system and the defect formation energies for a 15x15 SW defect and the for the *MikadoRR* and *BAFF* model. The datapoints correspond to the different scalings of the Mikado Model parameters.

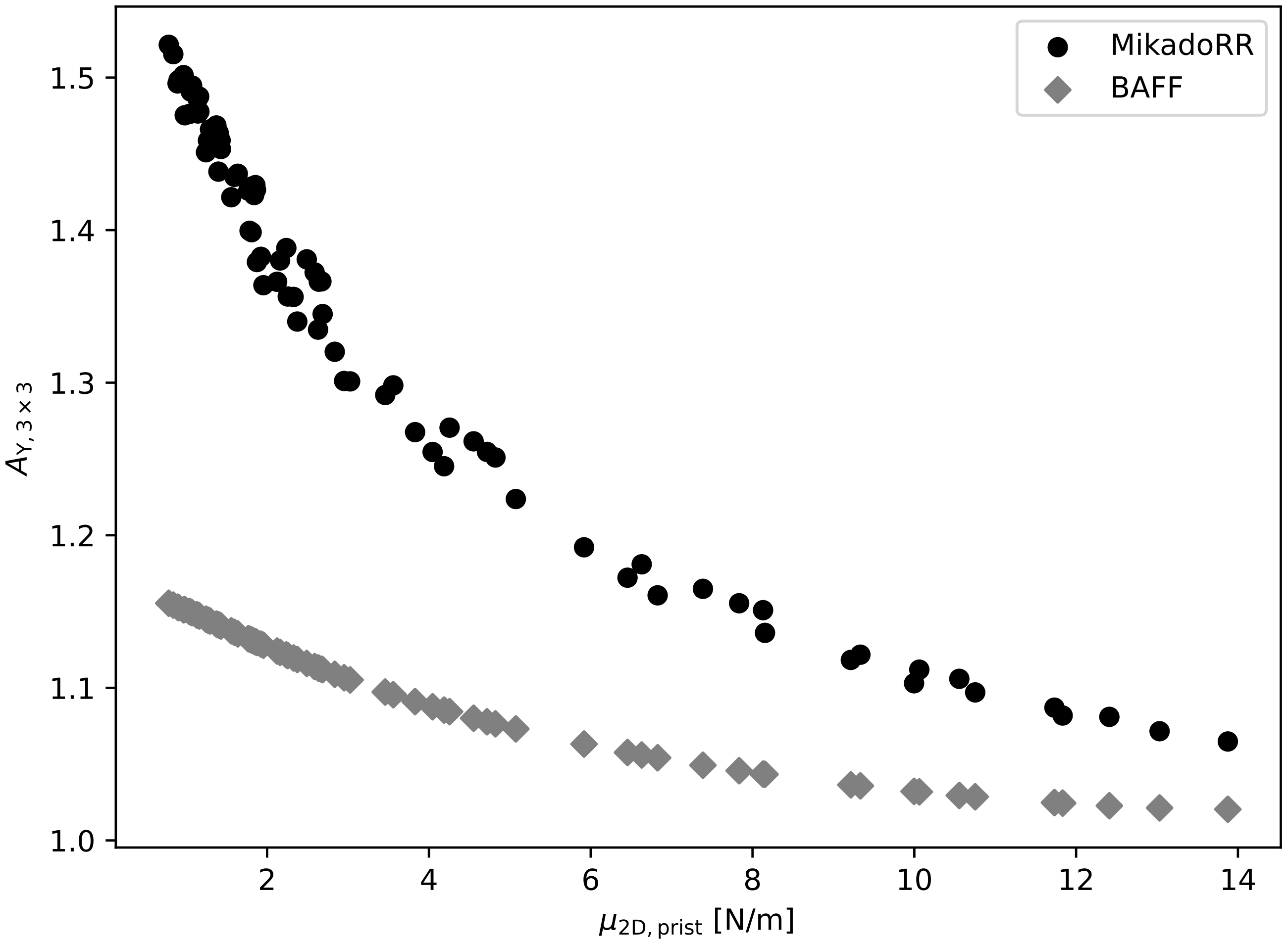

FIG. S15. Correlation between the shear modulus of the pristine system and anisotropy regarding the Young's modulus for a 3x3 SW defect and the for the *MikadoRR* and *BAFF* model. The datapoints correspond to the different scalings of the Mikado Model parameters.

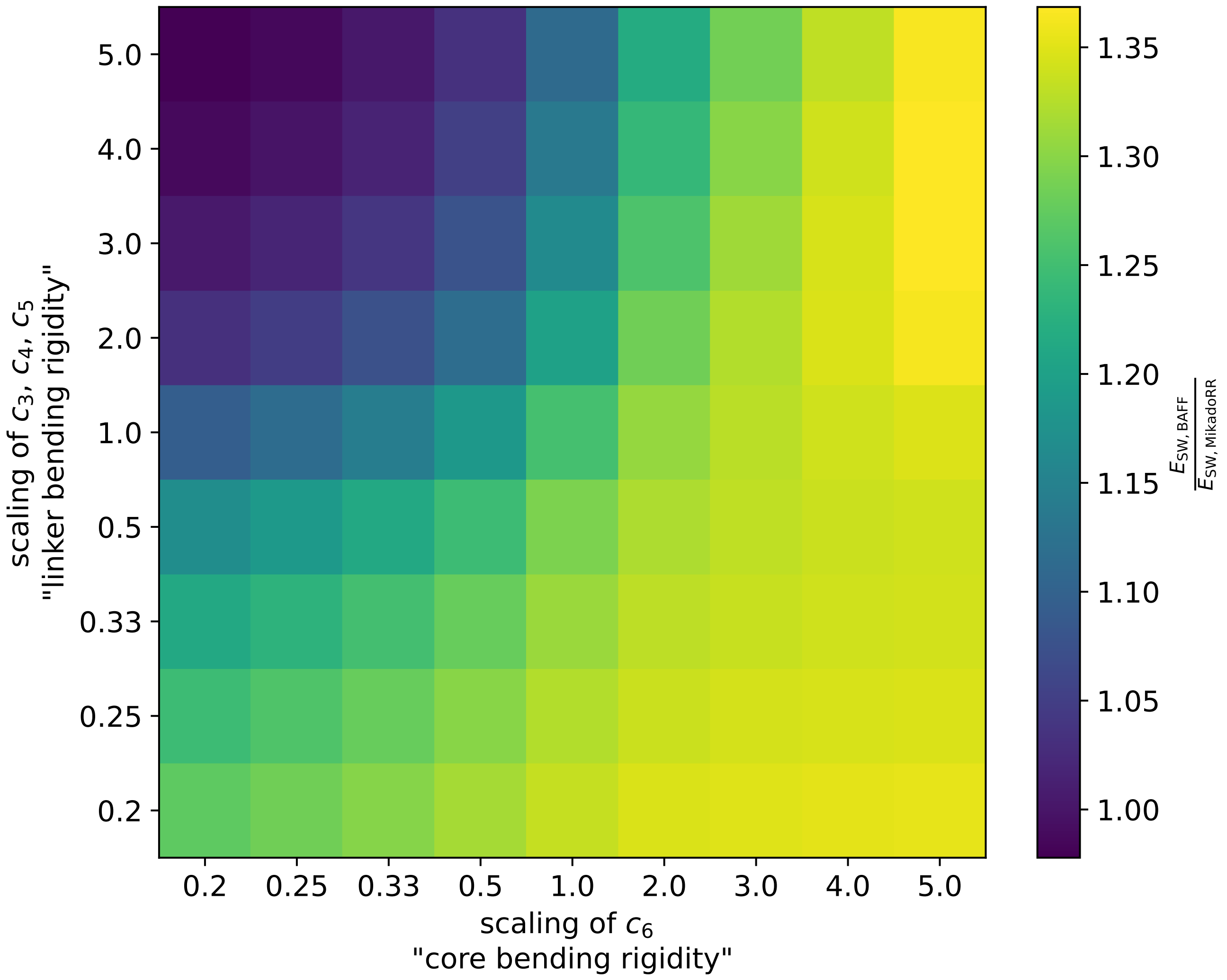

FIG. S16. The variation of the ratio of the defect formation energy of the *BAFF* and *MikadoRR* model in a 15x15 SW defect. The x- and y-axis represent the scaling of the parameter c_6 (corresponding to the core bending rigidity) and the parameters c_3, c_4 and c_5 (corresponding to the linker bending rigidity).

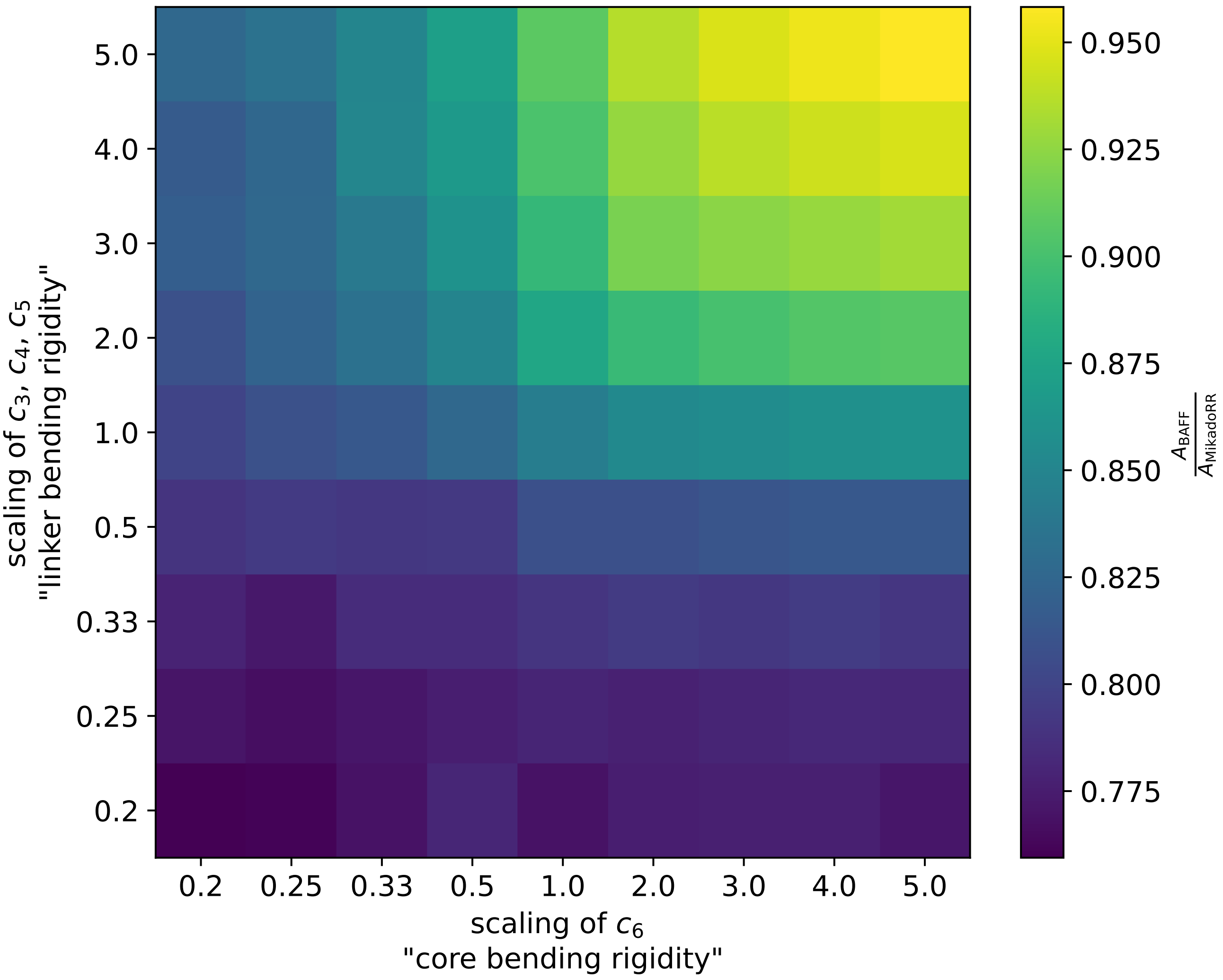

FIG. S17. The variation of the ratio of the anisotropy regarding the Young's modulus of the *BAFF* and *MikadoRR* model in a 03x03 SW defect. The x- and y-axis represent the scaling of the parameter c_6 (corresponding to the core bending rigidity) and the parameters c_3, c_4 and c_5 (corresponding to the linker bending rigidity).

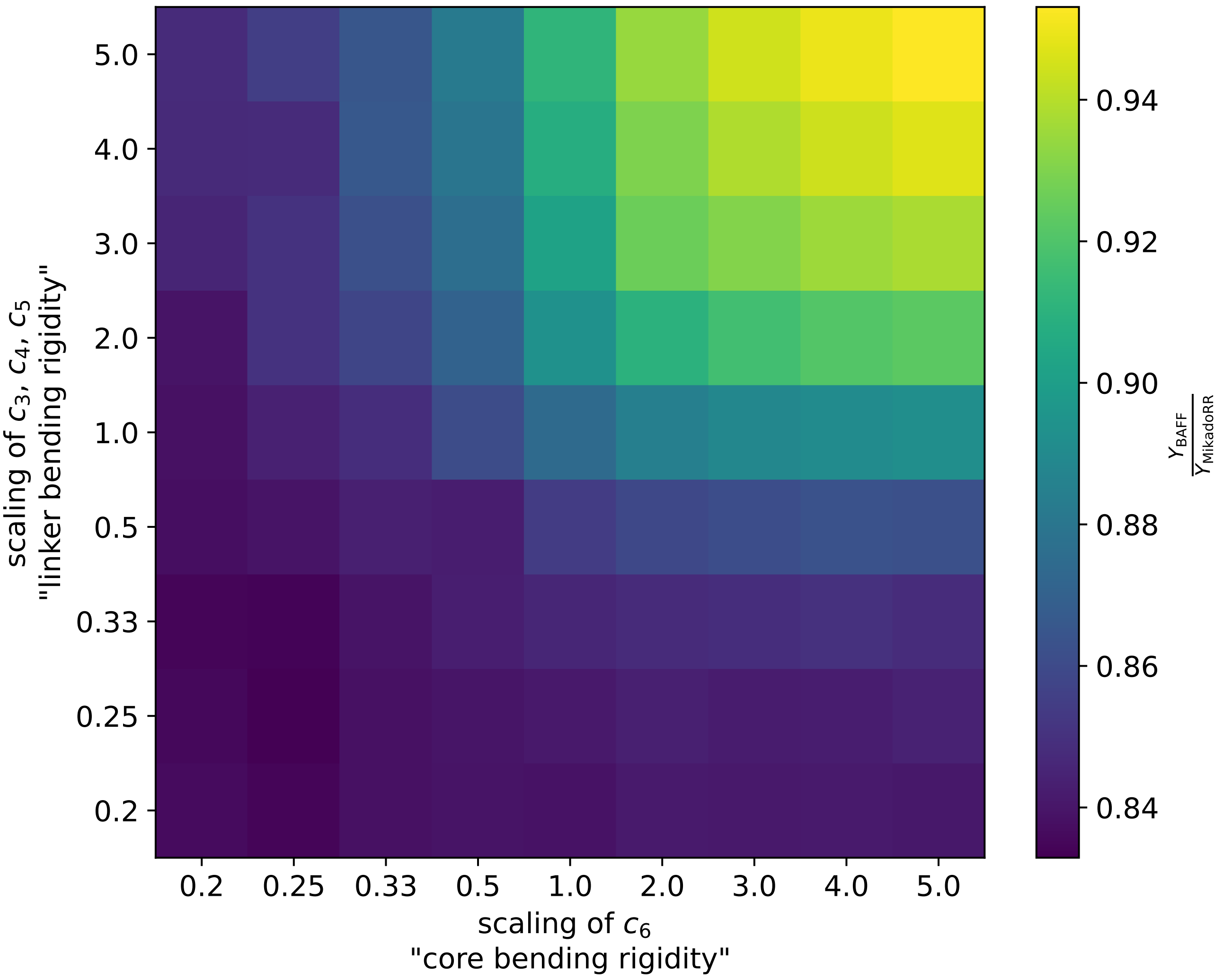

FIG. S18. The variation of the ratio of the maximum Young's modulus of the *BAFF* and *MikadoRR* model in a 03x03 SW defect. The x- and y-axis represent the scaling of the parameter c_6 (corresponding to the core bending rigidity) and the parameters c_3, c_4 and c_5 (corresponding to the linker bending rigidity).

A. Description of *MikadoRR*

The *MikadoRR* model describes the energy of the system based on per-beam contributions $\mathcal{H}_b$ and on per-node $\mathcal{H}_m$ contributions:

$$\mathcal{H}_{MikadoRR} = \sum_b \mathcal{H}_b + \sum_m \mathcal{H}_m \tag{S1}$$

$$\mathcal{H}_{\text{MikadoRR}} = \sum_b \left\{ c_1 d_b + c_2 d_b^2 + c_3 \psi_{b,0}^2 + c_4 \psi_{b,1}^2 + c_5 \psi_{b,0}\psi_{b,1} \right\} + c_6 \sum_m^N \sum_n^3 \phi_{mn}^2 + c_7 \ . \tag{S2}$$

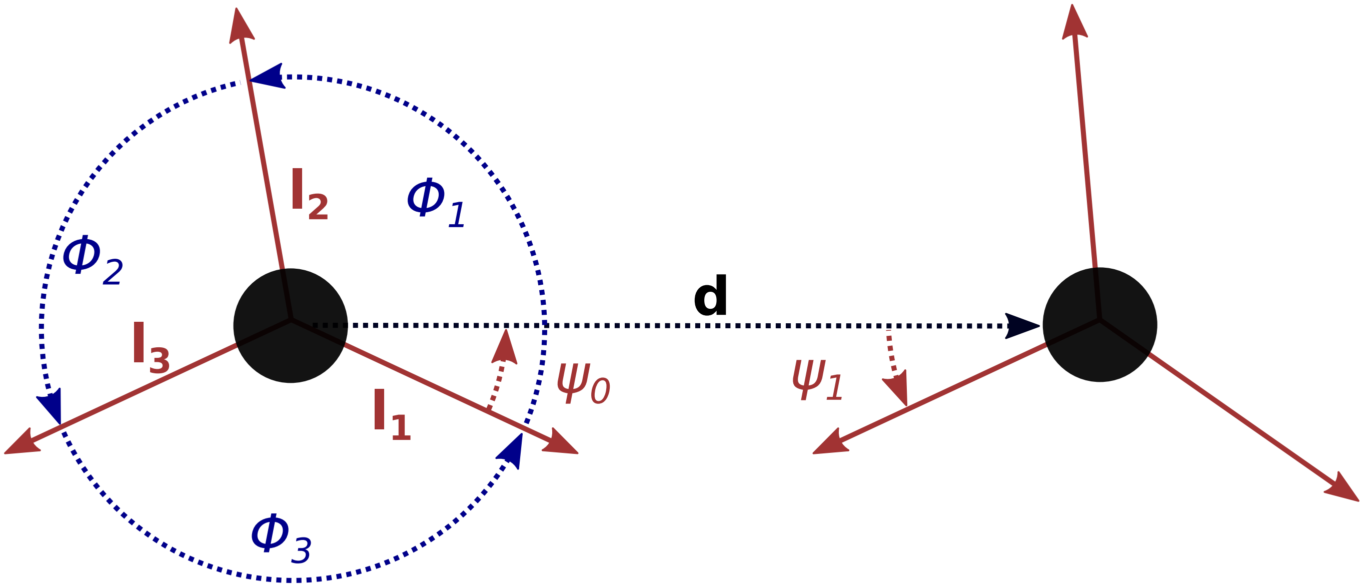

FIG. S19. Overview of the Mikado Model for one bond. Bold variables indicate vectors.

As shown in Fig. S19 ,the length d_b is the distance between the nodes connected by the beam. $\psi_{b,0}$ ($\psi_{b,1}$) is the angle between the vector from an incident node 0 (1) to a neighboring node 1 (0) and the linker-site vector $\mathbf{l}$ at node 0 (1). ϕ_{mn} is the angle at node m between two beams.

For the force-evaluation, the gradient of $\mathcal{H}_{\text{MikadoRR}}$ with respect to the core positions $\mathbf{r_i}$ must be evaluated. In the following, a short-hand notation is used where all variables are assumed to be related to site i or to i and j, e.g. $\mathbf{r_i}$ as $\mathbf{r}$, $\mathbf{d_{ij}}$ as $\mathbf{d}$, etc, unless explicitly written out. First, the distance-related features:

$$\frac{\partial|\mathbf{d}|}{\partial\mathbf{r}} = \frac{\partial\sqrt{\mathbf{d}\cdot\mathbf{d}}}{\partial\mathbf{r}} = \frac{\mathbf{d}}{|\mathbf{d}|} \ , \tag{S3}$$

$$\frac{\partial|\mathbf{d}|^2}{\partial\mathbf{r}} = \frac{\partial(\mathbf{d}\cdot\mathbf{d})}{\partial\mathbf{r}} = 2\mathbf{d} = 2|\mathbf{d}|\frac{\partial|\mathbf{d}|}{\partial\mathbf{r}} \ , \tag{S4}$$

with $\mathbf{d_{ij}} = \mathbf{r_j} - \mathbf{r_i}$ as the vector from core i to j.

The gradient of the angles Ψ:

$$\begin{aligned}
\frac{\partial\Psi}{\partial r_x} &= \frac{\partial\,\text{arctan2}(r_x l_y - r_y l_x, r_x l_x + r_y l_y)}{\partial r_x} \\
&= \frac{\partial\arctan(\frac{r_x l_y - r_y l_x}{r_x l_x + r_y l_y})}{\partial r_x} \\
&= \frac{1}{1+\frac{r_x l_y - r_y l_x}{r_x l_x + r_y l_y}} \frac{\partial \frac{r_x l_y - r_y l_x}{r_x l_x + r_y l_y}}{\partial r_x} \\
&= k^{\mathbf{d},\mathbf{l}}\left(\frac{\partial}{\partial r_x}\frac{r_x l_y - r_y l_x}{r_x l_x + r_y l_y}\right) \\
&= k^{\mathbf{d},\mathbf{l}}\left(\frac{-l_y}{r_x l_x + r_y l_y} + \frac{l_x(r_x l_y - r_y l_x)}{(r_x l_x + r_y l_y)^2}\right) \\
&= -k^{\mathbf{d},\mathbf{l}}\left(\frac{l_y}{\mathbf{l}\cdot\mathbf{d}} - \frac{l_x g^{\mathbf{d},\mathbf{l}}}{(\mathbf{l}\cdot\mathbf{d})^2}\right) \ .
\end{aligned} \tag{S5}$$

Here, $\mathbf{l_{ij}}$ is the linker-site vector of the linker at vertex i connecting a beam towards a linker-site at vertex j, which does not depend on the core position $\mathbf{r_i} = (r_{i,x}, r_{i,y})^T$. Hence, the derivative of Ψ^2 is:

$$\frac{\partial \Psi^2}{\partial r_x} = -2k^{\mathbf{d},\mathbf{l}} \left(\frac{l_y}{\mathbf{l}\cdot\mathbf{d}} - \frac{l_x g^{\mathbf{d},\mathbf{l}}}{(\mathbf{l}\cdot\mathbf{d})^2} \right) \Psi \ , \tag{S6}$$

$$\frac{\partial \Psi^2}{\partial r_y} = -2k^{\mathbf{d},\mathbf{l}} \left(-\frac{l_x}{\mathbf{l}\cdot\mathbf{d}} - \frac{l_y g^{\mathbf{d},\mathbf{l}}}{(\mathbf{l}\cdot\mathbf{d})^2} \right) \Psi \ . \tag{S7}$$

The core descriptors ϕ_{mi} do not depend on the position of the core and hence do not contribute to the gradient.

For the internal degrees of freedom, e.g. angles Ψ and ϕ, we derive a gradient which will be minimized for the energy evaluation. This is based on the derivative with respect to the linker-site vector $\mathbf{l_{ij}}$. The distance between vertices $|\mathbf{d}|$ is independent of $\mathbf{l_{ij}}$.

For the angles Ψ:

$$\frac{\partial \Psi}{\partial l_x} = \frac{\partial \arctan(\frac{r_x l_y - r_y l_x}{r_x l_x + r_y l_y})}{\partial l_x} = k^{\mathbf{d},\mathbf{l}} \left(-\frac{r_y}{\mathbf{l}\cdot\mathbf{d}} - \frac{r_x g^{\mathbf{d},\mathbf{l}}}{(\mathbf{l}\cdot\mathbf{d})^2} \right) \ . \tag{S8}$$

Hence, the derivative of Ψ^2 is:

$$\frac{\partial \Psi^2}{\partial l_x} = 2k^{\mathbf{d},\mathbf{l}} \left(-\frac{r_y}{\mathbf{l}\cdot\mathbf{d}} - \frac{r_x g^{\mathbf{d},\mathbf{l}}}{(\mathbf{l}\cdot\mathbf{d})^2} \right) \Psi \ , \tag{S9}$$

$$\frac{\partial \Psi^2}{\partial l_y} = 2k^{\mathbf{d},\mathbf{l}} \left(\frac{r_x}{\mathbf{l}\cdot\mathbf{d}} - \frac{r_y g^{\mathbf{d},\mathbf{l}}}{(\mathbf{l}\cdot\mathbf{d})^2} \right) \Psi \ . \tag{S10}$$

Angles ϕ are defined as the angles between two adjacent linker-sites $\mathbf{l_{ij}}$ and $\mathbf{l_{ik}}$ of a core. Hence, the derivative with respect to $\mathbf{l_{ij}}$ is:

$$\frac{\partial \phi}{\partial l_{ij,x}} = \frac{\partial \arctan(\frac{l_{ij,x} l_{ik,y} - l_{ij,y} l_{ik,x}}{l_{ij,x} l_{ik,x} + l_{ij,y} l_{ik,y}})}{\partial l_{ij,x}} = k^{\mathbf{l_{ij}},\mathbf{l_{ik}}} \left(\frac{l_{ik,y}}{\mathbf{l_{ij}}\cdot\mathbf{l_{ik}}} - \frac{l_{ik,x} g^{\mathbf{l_{ij}},\mathbf{l_{ik}}}}{(\mathbf{l_{ij}}\cdot\mathbf{l_{ik}})^2} \right) \ . \tag{S11}$$

The derivative of ϕ^2 is:

$$\frac{\partial \phi^2}{\partial l_{ij,x}} = 2k^{\mathbf{l_{ij}},\mathbf{l_{ik}}} \left(\frac{l_{ik,y}}{\mathbf{l_{ij}}\cdot\mathbf{l_{ik}}} - \frac{l_{ik,x} g^{\mathbf{l_{ij}},\mathbf{l_{ik}}}}{(\mathbf{l_{ij}}\cdot\mathbf{l_{ik}})^2} \right) \phi \tag{S12}$$

$$\frac{\partial \phi^2}{\partial l_{ij,y}} = 2k^{\mathbf{l_{ij}},\mathbf{l_{ik}}} \left(-\frac{l_{ik,x}}{\mathbf{l_{ij}}\cdot\mathbf{l_{ik}}} - \frac{l_{ik,y} g^{\mathbf{l_{ij}},\mathbf{l_{ik}}}}{(\mathbf{l_{ij}}\cdot\mathbf{l_{ik}})^2} \right) \phi \tag{S13}$$

B. Automatic feature extraction via beam-fit

To avoid as much human intervention as possible in defining the necessary characteristics for *MikadoRR*, we chose an approach that ignores the definition of what a core region and a linker region are. The only strictly necessary user input is the definition the geometric center of a core. The approach is based on fitting an elastic beam to the input geometry and extracting the features from the resulting beam equation.

1. Define center of cores via center of geometry (COG) of selected atoms

2. calculate the vector $\mathbf{d_{ij}}$ between the COGs

3. Define a linkage length l. Then create a corridor between two cores with width w and length $|\mathbf{d_{ij}}|-2l$. The corridor starts at $\mathbf{r_i} + l\frac{\mathbf{d_{ij}}}{|\mathbf{d_{ij}}|}$. All atoms within this corridor are selected.

4. Find Ψ_0 and Ψ_1 by minimizing the squared distance between the beam defined through the beam equation defining the orthogonal displacement y along x ($y = (x^2 - 2x + 1)x\Psi_0 + (x - 1)x^2\Psi_1$) and the atoms in the corridor.

5. Since we can calculate all ϕ from $\mathbf{d_{ij}}$ and all Ψ, we have extracted all the necessary characteristics.

By screening through several linkage lengths l and testing the performance of the model, a suitable l is selected which effectively describes what the core region of the COF is without user intervention. This avoids the ambiguous definition of what part of a COF is a core and what a linker is. The fitting procedure is discussed in C.

C. Fitting *MikadoRR*

The parameters c_1–c_7 are evaluated by fitting equation eq. S2 to energy-feature data. For the training data, in-plane isotropic strain is applied from -1.0% to 1.0% in steps of 0.1% and shearing strain is applied from -4.0% to 4.0% in steps of 0.4%. The strain is applied to the cell through a matrix $\mathbf{M}$ containing the scaling values the the cell vectors:

$$\mathbf{M_{iso}} = \begin{pmatrix} 1+s & 1+s & 0 \\ 1+s & 1+s & 0 \\ 0 & 0 & 1 \end{pmatrix} , \mathbf{M_{shear}} = \begin{pmatrix} 1+s & 1-s & 0 \\ 1+s & 1-s & 0 \\ 0 & 0 & 1 \end{pmatrix} , \tag{S14}$$

$$\mathbf{C_{strained}} = \mathbf{C} \odot \mathbf{M} , \tag{S15}$$

where $\mathbf{C}$ is the matrix containing the cell-vectors and $\odot$ is the element-wise matrix product. This results in 41 datapoints in total (20 with isotropic strain, 20 with shearing strain, one relaxed structure).

For each strained structure, the atomic positions are relaxed and the energy calculated. Then, the features are extracted as described in B. Based on the energies and features, a ridge regression with built-in cross-validation with a k-fold of $n_{datapoints}/3$ (i.e. 13 folds for 41 datapoints) as implemented in *scikit-learn* was used to determine parameters c_1–c_7. Multiple regularization strengths α are tested (1e-5 to 1e-12). The performance is evaluated through $R^2_{cv,best}$ which refers to the mean coefficient of determination (R^2) computed across all folds during cross-validation for the regularization strength α that yielded the highest score.

By training the model with different linker-lengths l, as outlined in B, the performance of the models is ranked according to the $R^2_{cv,best}$ score. Furthermore, trained models for which any of the $c_n = 0$, or $c_7 < 0$ or $c_3 < 0$ are discarded. This approach yields an optimal model with a linkage length l. All models for different linkage lengths are shown in table S1.

TABLE S1. Regression results for different linkage lengths. The bold-faced model is the optimal one based on the criteria described above.

linkage length [Å]	$R^2_{cv,best}$	R^2	RMSE [meV]	α	c_1	c_2	c_3	c_4	c_5	c_6	c_7
0.100	0.998401	0.999952	1.136225	1e-07	-49.8719	1.4006	7.0736	7.0736	7.0736	-19.3111	443.9425
0.403	0.998747	0.999989	0.545161	1e-12	-49.9328	1.4023	7.4733	7.4733	7.4575	-22.1109	444.4847
0.707	0.998515	0.999961	1.028744	1e-12	-49.9142	1.4018	7.1676	7.1674	7.1541	-20.0496	444.3189
1.010	0.998741	0.999989	0.546972	1e-12	-49.9342	1.4024	8.4094	8.4093	8.3933	-33.1589	444.4970
1.314	-1.816087	0.734144	84.765305	1e-07	-49.6563	1.3948	14.6181	14.6181	14.6182	-83.8890	441.9447
1.617	0.998767	0.999989	0.536056	1e-12	-49.9260	1.4022	9.0561	9.0561	9.0405	-47.1229	444.4240
1.921	0.998031	0.999952	1.134429	1e-12	-49.8818	1.4009	9.6617	9.6616	9.6562	-70.1717	444.0306
2.224	0.998504	0.999983	0.679783	1e-12	-49.9666	1.4033	10.1465	10.1469	10.1258	-104.7990	444.7853
2.528	-0.257899	0.997068	8.902449	1e-05	-44.0554	1.2373	9.9114	9.9114	9.9123	7.2732	392.1690
2.831	0.998480	0.999985	0.631242	1e-12	-49.9672	1.4033	10.8013	10.8012	10.7728	-298.6827	444.7905
3.135	0.995018	0.999602	3.278752	1e-09	-49.9172	1.4019	11.2203	11.2203	11.2203	548.4806	444.3450
3.438	0.998765	0.999988	0.559304	1e-10	-49.9029	1.4015	11.8652	11.8652	11.8651	168.7516	444.2183
3.742	0.998697	0.999986	0.610839	1e-12	-49.9116	1.4018	12.3424	12.3424	12.3291	103.4066	444.2955
4.045	0.998813	0.999990	0.525670	1e-12	-49.9190	1.4020	12.8741	12.8741	12.8509	78.3594	444.3616
4.349	0.998779	0.999990	0.530200	1e-09	-49.8836	1.4010	13.4880	13.4880	13.4880	60.8655	444.0467
4.652	0.998802	0.999990	0.523648	1e-10	-49.8856	1.4010	14.2968	14.2968	14.2967	47.6841	444.0639
4.956	0.988532	0.999760	2.549125	1e-06	-49.6045	1.3931	17.5505	17.5505	17.5505	28.5472	441.5617
5.259	0.998800	0.999990	0.528940	1e-12	-49.9221	1.4020	15.6996	15.6996	15.6658	35.6945	444.3888
**5.563**	**0.998823**	**0.999989**	**0.537751**	**1e-07**	**-49.8600**	**1.4003**	**16.7568**	**16.7568**	**16.7568**	**30.5518**	**443.8363**
5.866	-7.288203	0.864379	60.542234	1e-12	-38.6642	1.0860	-0.2981	-0.2971	6.7147	-2.5265	344.1476
6.170	-20.957432	0.544608	110.939863	1e-12	-33.2144	0.9332	-1.9375	-1.9363	9.7413	-2.3165	295.5469
6.473	-56.524352	0.243274	143.009197	1e-09	-29.0290	0.8160	-18.7539	-18.7539	39.8164	-0.6611	258.2061
6.777	-35.677631	0.292333	138.295860	1e-12	-30.2628	0.8506	-3.4139	-3.4140	10.0728	-0.8530	269.2025
7.080	-4.131322	0.792527	74.881622	1e-12	-43.8624	1.2320	1.7456	1.7461	5.7111	-3.8977	390.3932
7.384	-12.570224	0.657482	96.213538	1e-09	-38.0438	1.0688	-31.8146	-31.8146	72.8832	-4.0034	338.5646
8.294	-8056.014339	0.997772	7.760550	1e-05	-44.6119	1.2529	-8.8465	-8.8465	-8.8465	10.8961	397.1222
8.598	0.998486	0.999958	1.069522	1e-07	-49.8977	1.4014	-0.0000	-0.0000	-0.0000	11.1383	444.1724
8.901	0.998486	0.999958	1.069522	1e-07	-49.8977	1.4014	-0.0000	-0.0000	-0.0000	11.1383	444.1724

To evaluate the data efficiency of the method, we reduced the number of data points used for training step by step and followed the same procedure described above. The optimal training models for different number of data points are shown in table S2. The performance of the model does not reduce significantly for fewer number of data points.

TABLE S2. Regression results for different number of data points for training.

# data points	linkage length [Å]	$R^2_{cv,best}$	R^2	RMSE [meV]	α	c_1	c_2	c_3	c_4	c_5	c_6	c_7
41	5.563	0.998823	0.999989	0.537751	1e-07	-49.8600	1.4003	16.7568	16.7568	16.7568	30.5518	443.8363
21	3.438	0.999524	0.999990	0.524149	1e-11	-49.8148	1.3990	11.8637	11.8637	11.8637	168.4985	443.4333
11	4.652	0.998330	0.999995	0.393390	1e-12	-49.6219	1.3936	14.2611	14.2611	14.2751	47.8762	441.7133
9	5.563	0.998599	0.999997	0.333899	1e-09	-49.5282	1.3910	16.0575	16.0575	16.0576	31.6954	440.8764

D. Fitting *BAFF* via Equation of state

To fit the 2D bulk modulus B_{2D}, a third degree polynomial $E = a + bA + cA^2 + dA^3$ was fitted to energy E vs area A data. Hence, the bulk modulus is:

$$\frac{\partial E}{\partial A} = b + 2cA + 3dA^2 \overset{!}{=} 0 \; , \tag{S16}$$

$$A_{\mathrm{opt}} = \frac{-2c + \sqrt{4c^2 - 12bd}}{6d} \; , \tag{S17}$$

$$B_{\mathrm{2D}} = A_0 \frac{\partial^2 E}{\partial A^2}\Big|_{A=A_{\mathrm{opt}}} = A_0\left(2c + 6dA\right) = A_0\left(2c + 6d\frac{-2c + \sqrt{4c^2 - 12bd}}{6d}\right) = 2A_0\sqrt{c^2 - 3bd} \; . \tag{S18}$$

For the 2D shear modulus μ_{2D} energy vs shearing strain s data for the sheared structures was fitted:

$$\mu_{2D} = \frac{1}{4A_0} \frac{\partial^2 E}{\partial s^2}\Big|_{s=s_{opt}} = \frac{1}{2A_0}\sqrt{c^2 - 3bd} \;. \tag{S19}$$

E. Redecorating

As it is convenient to switch from a CG representation to an atomistic one, a redecoration functionality of the CG structure was implemented. First, the user defines molecular fragments of a linker and/or core (it is possible to either only use as core fragment, only a linker fragment or both). If a core is defined, it will be placed on the nodes of the CG-structure and rotated so that it most closely aligns to the neighboring nodes. If a linker is defined, it is placed at a predefined distance from the node and bent according to the elastic beam connecting two neighboring nodes.

[SI1] M. Elstner, D. Porezag, G. Jungnickel, J. Elsner, M. Haugk, T. Frauenheim, S. Suhai, and G. Seifert, Self-consistent-charge density-functional tight-binding method for simulations of complex materials properties, Physical Review B **58**, 7260–7268 (1998).

[SI2] M. Elstner, Scc-dftb: What is the proper degree of self-consistency?, The Journal of Physical Chemistry A **111**, 5614–5621 (2007).

[SI3] B. Lukose, A. Kuc, J. Frenzel, and T. Heine, On the reticular construction concept of covalent organic frameworks, Beilstein Journal of Nanotechnology **1**, 60–70 (2010).

[SI4] E. Caldeweyher, C. Bannwarth, and S. Grimme, Extension of the d3 dispersion coefficient model, The Journal of Chemical Physics **147**, 10.1063/1.4993215 (2017).
